# Investigation of Embedded FBG Response in Polymer Composites: Strain Transfer Mechanism and Interfacial Damage Under Multiaxial Loading

**DOI:** 10.3390/polym18151833

**Published:** 2026-07-27

**Authors:** Xingchen Yu, Zihe Cao, Dongfeng Cao, Haixiao Hu, Hongda Chen, Wei Cai, Shuxin Li

**Affiliations:** 1 State Key Laboratory of Materials Synthesis and Processing, Wuhan University of Technology, Wuhan 430070, China; yxc199602024499@163.com (X.Y.); 220482@whut.edu.cn (Z.C.); cao_dongf@whut.edu.cn (D.C.); hongdachen@whut.edu.cn (H.C.); caiwei199696@whut.edu.cn (W.C.); 2 National Energy Key Laboratory for New Hydrogen-Ammonia Energy Technologies, Foshan Xianhu Laboratory, Foshan 528000, China; 3 Institute for Frontier and Interdisciplinary Innovation (School of Future Technology), Wuhan University of Technology, Wuhan 430119, China

**Keywords:** composite laminates, embedded FBGs, strain transfer, FE modelling

## Abstract

Fiber Bragg gratings (FBGs) embedded in polymer composite materials is a promising method for structural health monitoring (SHM). Conventionally, FBGs are treated as axial strain sensors with a constant sensitivity coefficient, which can lead to significant measurement errors for the multi-axial stress states. This study developed a general framework to calculate the axial strain sensitivity coefficient considering the local multiaxial strain state of the host composite materials, where the analytical strain transfer principle (STP) was integrated with the Opto-mechanical model of FBGs. The responses of embedded FBGs under hydrostatic pressure, biaxial compression, and biaxial tension conditions were validated by the experimental tests. The results further reveal the strong influence of the interfacial bonding state between the FBG and host composite material on the FBG response. This study advances the embedded FBG technology in SHM for composites.

## 1. Introduction

The outstanding specific modulus, specific strength, and formability of advanced carbon fiber-reinforced polymers (CFRPs) have enabled their extensive adoption across high- aerospace [1,2], pressure vessels [3] and marine engineering [4]. In civil aviation, weight re-duction directly translates into improved payload and fuel efficiency, which has driven the deployment of polymer composites from secondary parts to primary load-bearing structures such as wing boxes. However, polymer composites are prone to internal defects and damage during both manufacturing and service due to process variations and com-plex static or fatigue loads [5]. Their anisotropic nature means damage often initiates and grows internally, making their detection difficult. Conventional offline nondestructive testing methods, including ultrasonic C-scans and computed tomography, cannot provide continuous monitoring of in-service structures. To enable real-time condition assessment, the integration of sensors into polymer composite structures has emerged as a practical strategy for structural health monitoring (SHM) [6].

Traditional electrical sensors suffer from zero drift, nonlinear response, electromagnetic interference, and complex wiring. Fiber-optic sensors, in contrast, offer immunity to electromagnetic interference, support multiplexing, and are compact and embeddable within polymer matrices [7]. Among these, wavelength-modulated fiber Bragg grating (FBG) sensors use Bragg wavelength encoding for wavelength-division multiplexing (WDM), enabling real-time multipoint monitoring of strain, temperature, and pressure. Because of their small size, durability, and reliability, FBG sensors are promising to be used in aerospace [8], marine engineering [9], wind energy [10], automotive [11], pressure vessels [12], and composite manufacturing [13]. Compared with surface-mounted counterparts, embedded FBGs measure internal strain, pick up less environmental noise, and enable continuous monitoring from manufacturing through service. Embedded FBGs have caused widely attention to monitor curing [14,15], thermal [16] and hygroscopic [17] expansion, quasi-static loading [18], interlaminar damage [19], and low-velocity impacts [20] in polymer composites.

Despite their widespread use, embedded FBGs are often treated as axial strain sensors, with temperature compensation applied, where axial strain is calculated using the theoretical strain sensitivity coefficient of a bare FBG under axial stress (about 1.22 pm/με). It ignores the multiaxial stress states found in critical regions of polymer composites. The axial stress assumption works for surface-bonded FBGs because lateral constraint is minimal. For embedded FBGs that experience multidirectional constraints and complex strain transfer from the surrounding host [21,22], the assumption fails. This limitation also complicates damage detection. Although axial strain gradients from damage can produce chirped spectra [23], the spectral distortions observed in embedded FBGs are not always well explained by this effect alone [24]. Transverse stresses induce birefringence, which likely adds to the distortion. Analyses that attribute such spectral changes solely to axial gradients therefore risk mixing up the two mechanisms and overlooking the contribution of the multiaxial stress field.

Bare FBGs are sensitive to transverse stresses [25] and hydrostatic pressures [26]. Photoelastic theory can explain this behavior [27]. FBGs are inherently sensitive to both axial and non-axial stresses, producing measurable spectral changes including wavelength shifts and birefringence. When embedded, they retain this sensitivity and can sense multiaxial stress variations from deformation of the surrounding host, rather than responding only to axial stress. Bosia et al. [21] observed spectral broadening and splitting in an epoxy-embedded FBG subjected to biaxial compression. Lai et al. [22] reported complex multiaxial spectral responses from embedded FBGs under biaxial compression and hydrostatic pressure. Beyond fundamental characterization, Albero Blanquer et al. [28] used the transverse stress sensitivity of an embedded FBG to monitor stress evolution at the electrode-electrolyte interfaces in lithium-ion batteries.

When embedded in polymer composites, FBGs exhibit birefringence that contains multiaxial strain information. Minakuchi et al. [29] embedded an FBG in an L-shaped composite part and used birefringence to monitor through-thickness strain changes over the structural life cycle, capturing key indicators of spring-in distortion during manufacturing and through-thickness tensile failure in operation. Takagaki et al. [30] employed FBG-based monitoring to evaluate radial strain development in thick-walled CFRP pipes during curing, characterizing residual stress evolution and validating a stress-reduction method for crack-free fabrication. Wachtarczyk et al. [31] used highly birefringent FBGs inscribed in side-hole elliptical core fiber to measure in-plane and out-of-plane residual strains during manufacturing, as well as transverse strain behavior under cyclic loading in thermoplastic composites. These studies show that embedded FBGs produce a multiaxial response under complex stress states, a response that a purely axial assumption model cannot account for. However, while some researchers have refined calibration coefficients [32,33] or utilized birefringence as an indicator of multiaxial strain, a fundamental gap remains. Recently, Chen et al. [34] and Zhou et al. [35] made progress by incorporating the strain transfer principle (STP) into multi-directional strain measurement using embedded FBGs. Chen et al. [34] employed an isotropic polyetherimide (PEI) as the host material, while Zhou et al. [35] used Carbon Fiber/PEI cross-ply laminates. Despite these different material systems, both studies share a common assumption that the in-plane strains parallel and perpendicular to the FBG axis in the host material are equal. Moreover, the physical mechanisms governing the interaction between an embedded FBG and the surrounding matrix are still not sufficiently quantified or understood [36,37].

To address the gap between opto-mechanical model and multiaxial strain states in composites, the present study develops an integrated analytical framework based on the STP [38]. The framework quantifies how multiaxial strains from the host material are transmitted to the embedded FBG using a strain transfer coefficient (STC) matrix. This matrix maps the full multiaxial strain field of the host at the embedding location to the strain state experienced by the FBG. The framework is implemented as follows. First, comparative numerical modeling is performed for key multiaxial loading conditions (hydrostatic pressure [22], biaxial tension [39], biaxial compression [21]) in host materials including epoxy and CFRP. Strain fields from models with and without an embedded FBG are compared to directly quantify strain transfer. Then, the strain field from the no-FBG model is fed through the integrated STP and opto-mechanical model to predict the FBG spectral response. These predictions are validated against literature reported experimental measurements. Unlike prior approaches [34,35], the present study extends the STP to more general loading conditions involving different strain components in the host material. For the present approach, the variation in the apparent axial strain sensitivity coefficient of embedded FBGs in polymer composites under various loading conditions is considered. The theoretical model is established under the assumption of perfect interfacial bonding; interfacial damage is not incorporated into the primary predictive framework. Instead, this study semi-quantitatively analyze its influence in the biaxial tension subsection by systematically varying the coefficients of the strain STC matrix, aiming to interpret experimental–theoretical discrepancies and assess its impact on strain transfer and the FBG signal response. By combining comparative modeling, the STP, and opto-mechanical model, this work clarifies how embedded FBGs respond under realistic CFRP loading, aiding SHM applications.

## 2. Working Principle of Embedded FBG Sensing

An FBG is fabricated by inscribing a permanent, periodic or semi-periodic modulation of the refractive index along the fiber core. Within the grating region, the spatial modulation of the refractive index is described by [40]:

(1) $$\Delta n\left(x\right)=\bar{\Delta n\left(x\right)}\left[1+\nu \cos\left(\frac{2\pi }{\Lambda }x+\varphi \left(x\right)\right)\right]$$

where $\bar{∆n(x)\;}$ is the spatially averaged modulation depth per period, *ν* is the fringe visibility (typically ~1 for strong gratings), Λ is the grating period, and *φ*(*x*) determines how the grating period changes along the fiber (with *x* along the fiber axis). For uniform FBGs, *φ*(*x*) is constant, giving an unchirped initial reflection spectrum.

**Figure 1:**
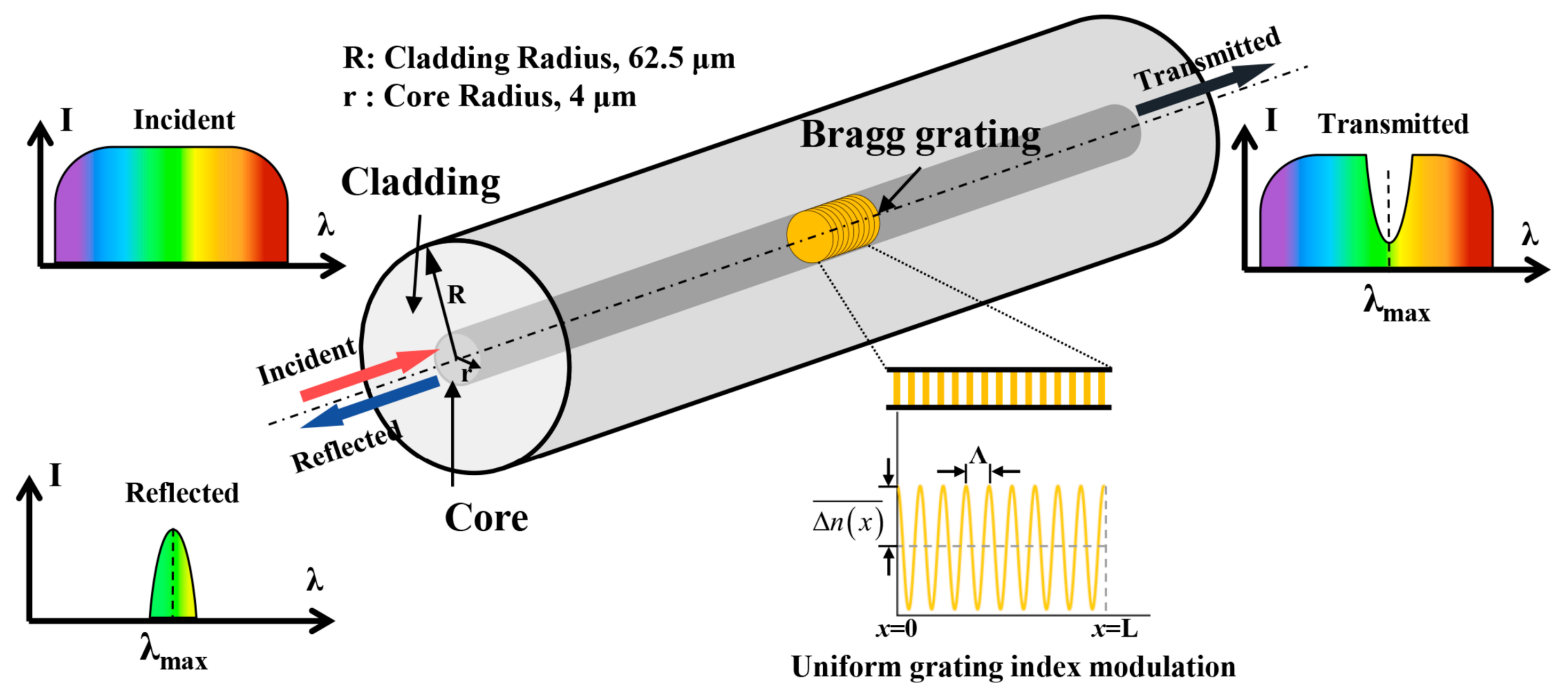
Schematic of FBG sensing with uniform index modulation.

For a uniform grating (Figure 1), the type used in this study, the phase fronts are perpendicular to the fiber axis, and the grating has a constant period Λ. Broadband light traveling through the grating is scattered by each plane.

For most wavelengths, the reflected light from successive planes gradually falls out of phase and cancels out. At a specific wavelength satisfying the Bragg condition, the reflections from all planes add constructively in the backward direction, producing a strong back-reflected peak. In transmission, this resonance appears as a distinct dip. The central wavelength of this resonance, the Bragg wavelength, is given by:

(2) $$\lambda_{B}=2n_{eff}\Lambda$$

here, *λ_B_* is the free-space center wavelength of the light that is back-reflected from the Bragg grating, *n_eff_* is the effective refractive index of the fiber core at this wavelength.

Equation (2) defines the Bragg wavelength in the reference state (free of mechanical and thermal perturbations). When a bare uniform FBG is subjected to uniform axial stress or temperature variation, both the grating period Λ and the effective refractive index *n_eff_* change, causing a shift in *λ_B_*. This shift forms the basis for conventional FBG-based strain and temperature sensing.

A non-uniform axial strain field along a uniform FBG can distort the reflected spectrum through chirping. For the short FBGs used in this study (≤5 mm), however, strain variation along the grating remains small under most conditions, so conventional wavelength shift analysis remains valid. Detailed treatment of chirp-induced distortions is beyond the scope of this work. On this basis, the sensing model for an embedded FBG is conventionally simplified to assume that it experiences only temperature and axial mechanical strain. The simplification yields the following linear relationship between the Bragg wavelength shift Δ*λ*_B_, axial strain *ε*, and temperature change Δ*T*.

(3) $$\Delta \lambda_{B}=K_{\varepsilon }\Delta \varepsilon +K_{T}\Delta T$$

In Equation (3), *K_ε_* and *K_T_* are the sensitivity coefficients for axial strain and temperature, respectively. This linear relationship serves as the model for embedded FBG applications in composite SHM [9,13].

This common approach treats the embedded FBG as a temperature-compensated axial strain gauge, neglecting its complex interaction with the host under multiaxial stress. A comprehensive theoretical framework is needed to establish the basis for FBG responses under multiaxial loading, accounting for the full three-dimensional strain field in the host. Such a framework should capture both the intrinsic optomechanical response of the bare FBG and the mechanics of strain transfer across the embedded interface, without relying on a priori simplification about the stress state.

The present work begins with the FBG mode coupling equations and combines optomechanical coupling with strain transfer theory to uncover the physical origins of the signal response.

### 2.1. Mode Coupling Mechanism

The periodic refractive index modulation in an FBG perturbs light propagation. This perturbation couples energy between the forward and backward-propagating components of the fundamental fiber mode. The coupling strength determines the reflection power *R* of the grating.

(4) $$\begin{array}{l} R=\frac{\sinh^{2}\left(\sqrt{\kappa^{2}-{\hat{\sigma }}^{2}}L\right)}{\cosh^{2}\left(\sqrt{\kappa^{2}-{\hat{\sigma }}^{2}}L\right)-\frac{{\hat{\sigma }}^{2}}{\kappa^{2}}}\\ where\left\{\begin{array}{l} \hat{\sigma }=\sigma +\delta -\frac{1}{2}\frac{d\varphi \left(x\right)}{dx}=\left(\frac{2\pi }{\lambda }\bar{\Delta n(x)}\right)+\left(\beta_{1}-\frac{\pi }{\Lambda }\right)-\frac{1}{2}\frac{d\varphi \left(x\right)}{dx}\\ \kappa =\frac{\sigma \nu }{2}=\frac{1}{2}\left(\frac{2\pi }{\lambda }\bar{\Delta n(x)}\right)\nu \end{array}\right. \end{array}$$

here, *λ* is the propagating wavelength, *σ* is the direct current (DC) self-coupling coefficient, and *κ* is the alternating current (AC) coupling coefficient. The detuning parameter *δ* quantifies the phase mismatch between the incident wave and the grating period. Phase matching occurs when *δ* = 0. Substituting the propagation constant of the fundamental fiber mode, *β*_1_ = 2π*n_eff_*/*λ_B_*, into this condition recovers the classical Bragg condition (Equation (2)) [41].

For the uniform FBG used in this study, the fringe visibility ν, the refractive index modulation depth $\bar{∆n(x)}$, and the phase term *φ*(*x*) are all constant along the grating. Under these conditions, both the AC coupling coefficient *κ* and the DC self-coupling coefficient $\hat{\sigma }$ depend on the effective refractive index *n_eff_* and the grating period Λ.

The FBG supports two orthogonal polarization modes (fast and slow axes), each with its own effective refractive index. The overall reflection spectrum is the superposition of the responses from these two modes. The next section examines how each of these polarization-dependent refractive indices is modulated by mechanical and thermal fields through the elasto-optic and thermo-optic effects, thereby linking the multiaxial strain state to the observable spectral changes.

### 2.2. Opto-Mechanical Model

The mode coupling analysis in the previous section shows that the FBG reflection spectrum is governed by the effective refractive index *n_eff_*. This parameter is modulated by external fields through the photoelastic and thermo-optic effects. The quantitative relationship between changes in *n_eff_* and applied strain or temperature can be derived from the fundamental wave equation for light propagation in an optical waveguide [38]:

(5) $$\left\{\begin{array}{l} \Delta n_{eff,p}=-\frac{n_{eff,0}^{3}}{2}\left\{\begin{array}{l} p_{12}\varepsilon_{1}+\left(p_{11}+p_{12}\right)\frac{\varepsilon_{2}+\varepsilon_{3}}{2}-\left[\frac{2}{n_{eff,0}^{3}}\left(\frac{\partial n_{eff}}{\partial T}\right)+\left(p_{11}+2p_{12}\right)\alpha \right]\Delta T\\ +\frac{p_{11}-p_{12}}{2}\sqrt{{\left(\varepsilon_{2}-\varepsilon_{3}\right)}^{2}+\gamma_{23}^{2}} \end{array}\right\}\\ \Delta n_{eff,q}=-\frac{n_{eff,0}^{3}}{2}\left\{\begin{array}{l} p_{12}\varepsilon_{1}+\left(p_{11}+p_{12}\right)\frac{\varepsilon_{2}+\varepsilon_{3}}{2}-\left[\frac{2}{n_{eff,0}^{3}}\left(\frac{\partial n_{eff}}{\partial T}\right)+\left(p_{11}+2p_{12}\right)\alpha \right]\Delta T\\ -\frac{p_{11}-p_{12}}{2}\sqrt{{\left(\varepsilon_{2}-\varepsilon_{3}\right)}^{2}+\gamma_{23}^{2}} \end{array}\right\} \end{array}\right.$$

**Figure 2:**
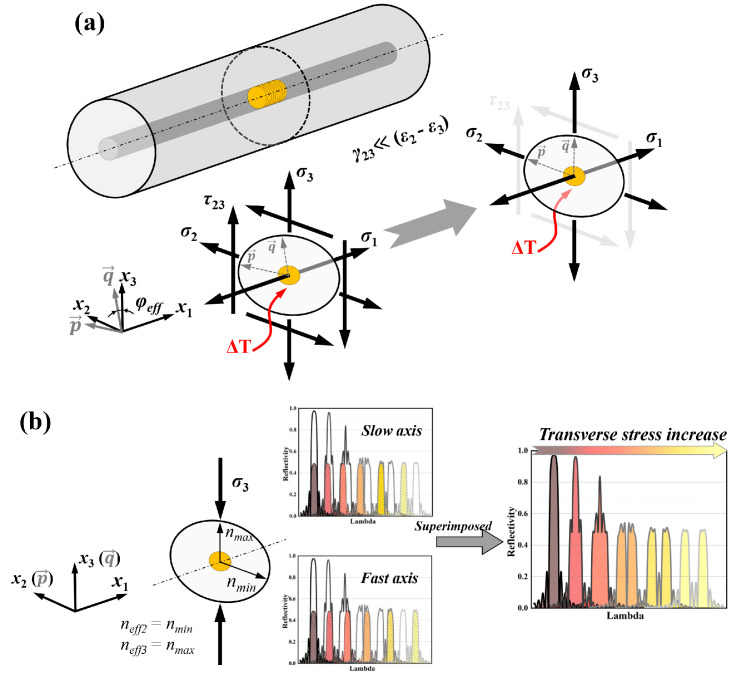
Bare FBG under multiaxial and transverse loading: (**a**) multiaxial uniform stress and temperature field; (**b**) out-of-plane transverse compressive stress inducing spectral broadening.

In Equation (5), *p_ij_* are components of the photoelastic tensor **P**, and *ε_j_* denotes the strain components within the fiber core. Vectors $\vec{p}$ and $\vec{q}$ represent two mutually orthogonal polarization directions in the fiber cross-section (Figure 2a). These directions coincide with the two principal strain directions, meaning they are oriented so that no shear strain *γ*_23_ is induced. The principal strain directions are determined by the applied multiaxial stress state. In the presence of *γ*_23_, they rotate relative to the fixed coordinate axes *x*_2_ and *x*_3_ by an angle *φ_eff_*. Consequently, $\vec{p}$ and $\vec{q}$ are not necessarily aligned with *x*_2_ and *x*_3_. The other shear components *γ*_13_ and *γ*_12_ are omitted because their effect is negligible. They would induce a minute tilt of the grating plane (~0.573° for an extreme shear of 10,000 με), causing no significant spectral change [41].

For embedded FBGs, the shear strain *γ*_23_ is typically much smaller in magnitude than (*ε*_2_ − *ε*_3_) and can be neglected. Thus, Equation (5) simplifies to:

(6) $$\begin{array}{l} \left\{\begin{array}{l} \Delta n_{eff,2}=-\frac{n_{eff,0}^{3}}{2}\left(p_{12}\varepsilon_{1,m}+p_{11}\varepsilon_{2,m}+p_{12}\varepsilon_{3,m}-\frac{2}{n_{0}^{3}}\left(\frac{\partial n_{eff}}{\partial T}\right)\Delta T\right)\\ \Delta n_{eff,3}=-\frac{n_{eff,0}^{3}}{2}\left(p_{12}\varepsilon_{1,m}+p_{12}\varepsilon_{2,m}+p_{11}\varepsilon_{3,m}-\frac{2}{n_{0}^{3}}\left(\frac{\partial n_{eff}}{\partial T}\right)\Delta T\right) \end{array}\right.\\ where\;\left\{\begin{array}{l} \Delta n_{eff,p}=\Delta n_{eff,2}\Delta n_{eff,q}=\Delta n_{eff,3}when\;\varepsilon_{2}>\varepsilon_{3}\\ \Delta n_{eff,q}=\Delta n_{eff,2}\Delta n_{eff,p}=\Delta n_{eff,3}when\;\varepsilon_{2}<\varepsilon_{3} \end{array}\right. \end{array}$$

where subscript *m* denotes mechanical strain within the FBG core (i.e., strain component independent of thermal expansion). Since this study focuses exclusively on mechanical loading under multiaxial stress states, temperature effects are not considered in the subsequent analysis. The thermo-optic coefficient *n_eff_*_,0_ × ∂*n*/∂*T* is denoted as *ξ* but is not activated in the present work. Equation (6) defines the dependence of *n_eff_* on the local thermo-mechanical fields, forming the basis for predicting the FBG spectral response under general loading conditions.

For short-gauge embedded FBGs, the strain distribution along the grating axis is typically uniform. This justifies the use of a “point-sensor” model under a multiaxial stress state (Figure 2a). In practice, however, this point-sensor model is often further simplified to a temperature-compensated axial strain gauge (Equation (3)). That simplification assumes pure axial stress and overlooks how multiaxial stress components in composites modulate *n_eff_*, leading to measurement errors.

To include multiaxial stress effects, the Bragg condition is expanded via a first-order Taylor series, giving a generalized expression coupling wavelength shift to strain and temperature.

(7) $$\frac{\Delta \lambda_{B}(\Delta \varepsilon_{i},\Delta T)}{\lambda_{B,0}}=\frac{\Delta n_{eff}(\Delta \varepsilon_{i},\Delta T)}{n_{eff,0}}+\frac{\Delta \Lambda }{\Lambda }$$

where *i* = 1, 2, 3, 4 and *ε*_4_ = *γ*_23_. Combining this with Equation (6) gives:

(8) $$\left\{\begin{array}{l} \frac{\Delta \lambda_{B,p}}{\lambda_{B,0}}=-\frac{n_{eff,0}^{2}}{2}\left[p_{12}\varepsilon_{1}+\left(p_{11}+p_{12}\right)\frac{\varepsilon_{2}+\varepsilon_{3}}{2}+\frac{p_{11}-p_{12}}{2}\sqrt{{\left(\varepsilon_{2}-\varepsilon_{3}\right)}^{2}+\gamma_{23}^{2}}+\left(p_{11}+2p_{12}\right)\alpha \Delta T\right]\\ \;\;\;\;+\varepsilon_{1}^{m}+(\alpha +\xi )\Delta T\\ \frac{\Delta \lambda_{B,q}}{\lambda_{B,0}}=-\frac{n_{eff,0}^{2}}{2}\left[p_{12}\varepsilon_{1}+\left(p_{11}+p_{12}\right)\frac{\varepsilon_{2}+\varepsilon_{3}}{2}-\frac{p_{11}-p_{12}}{2}\sqrt{{\left(\varepsilon_{2}-\varepsilon_{3}\right)}^{2}+\gamma_{23}^{2}}+\left(p_{11}+2p_{12}\right)\alpha \Delta T\right]\\ \;\;\;\;+\varepsilon_{1}^{m}+(\alpha +\xi )\Delta T \end{array}\right.$$

Neglecting the shear strain *γ*_23_ simplifies the expression to:

(9) $$\begin{array}{l} \left\{\begin{array}{l} \frac{\Delta \lambda_{B2}}{\lambda_{B}}=-\frac{n_{eff,0}^{2}}{2}(p_{12}\varepsilon_{1}^{m}+p_{11}\varepsilon_{2}^{m}+p_{12}\varepsilon_{3}^{m})+\varepsilon_{1}^{m}+\left(\alpha +\xi \right)\Delta T\\ \frac{\Delta \lambda_{B3}}{\lambda_{B}}=-\frac{n_{eff,0}^{2}}{2}(p_{12}\varepsilon_{1}^{m}+p_{12}\varepsilon_{2}^{m}+p_{11}\varepsilon_{3}^{m})+\varepsilon_{1}^{m}+\left(\alpha +\xi \right)\Delta T \end{array}\right.\\ where\;\left\{\begin{array}{l} \Delta \lambda_{B2}=\Delta \lambda_{B,p}\Delta \lambda_{B3}=\Delta \lambda_{B,q}when\;\varepsilon_{2}^{m}>\varepsilon_{3}^{m}\\ \Delta \lambda_{B2}=\Delta \lambda_{B,q}\Delta \lambda_{B3}=\Delta \lambda_{B,p}when\;\varepsilon_{2}^{m}<\varepsilon_{3}^{m} \end{array}\right. \end{array}$$

Under pure axial stress, transverse strains follow from Poisson’s ratio, and the model reduces to the conventional embedded FBG sensing expression (Equation (3)). Under a general multiaxial stress state, however, the effective refractive indices along the two principal polarization axes change by different amounts, causing birefringence. This birefringence creates a wavelength separation between the two polarization modes. When the separation exceeds a threshold, the single reflection peak broadens and then splits into two distinct peaks.

Transverse compressive stress is a typical case. It induces birefringence through differential transverse strains, driving the spectral evolution from broadening to full splitting (Figure 2b). Under such conditions, monitoring only the central wavelength is insufficient. To describe the spectral evolution, two parameters are introduced: the average spectral shift Δ*λ_avg_* and the spectral broadening Δ*λ_diff_*, defined as the wavelength separation between the fast- and slow-axis reflection peaks:

(10) $$\left\{\begin{array}{l} \frac{\Delta \lambda_{avg}}{\lambda_{B}}=\frac{\Delta \lambda_{B2}+\Delta \lambda_{B3}}{2\lambda_{B}}=-\frac{n_{eff,0}^{2}}{2}\left[p_{12}\varepsilon_{1}^{m}+\frac{p_{11}+p_{12}}{2}\left(\varepsilon_{2}^{m}+\varepsilon_{3}^{m}\right)\right]+\varepsilon_{1}^{m}+\left(\alpha +\xi \right)\Delta T\\ \frac{\Delta \lambda_{diff}}{\lambda_{B}}=\left|\frac{\Delta \lambda_{B2}-\Delta \lambda_{B3}}{\lambda_{B}}\right|=\left|\frac{n_{eff,0}^{2}}{2}\left(p_{11}-p_{12}\right)\left(\varepsilon_{2}^{m}-\varepsilon_{3}^{m}\right)\right| \end{array}\right.$$

Determining these parameters accurately requires balanced excitation of both polarization axes. This is achieved with an unpolarized broadband light source to avoid weighting bias in the reflected spectra.

### 2.3. Strain Transfer Principle

**Figure 3:**
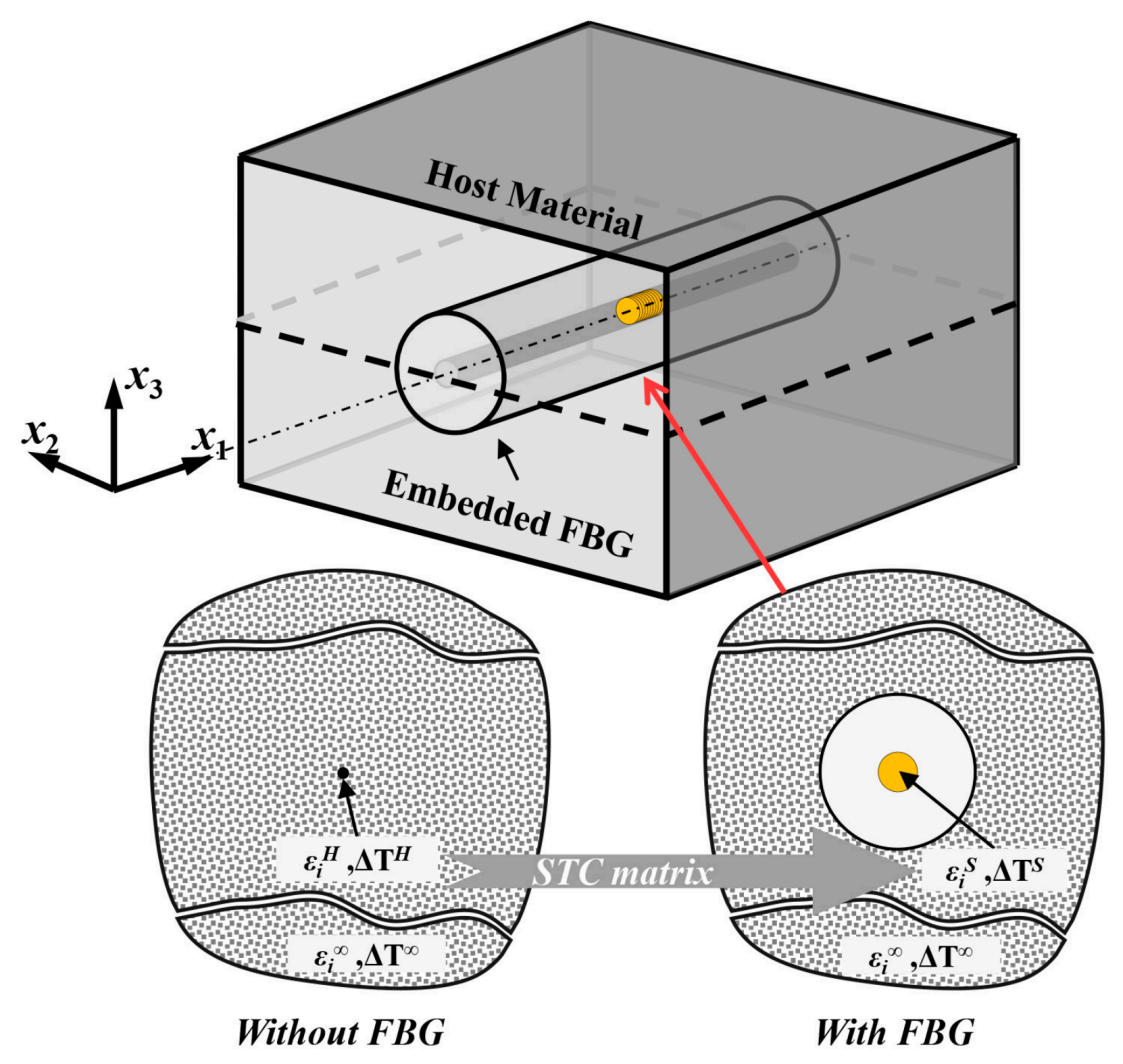
Strain transfer in host material due to embedded FBG sensor.

The theory above relates the FBG spectral response to the strain and temperature of the localized FBG. For an embedded FBG, strain transfers from the host to the FBG. The mismatches of elastic modulus, Poisson’s ratio, and thermal expansion coefficient would perturb the local strain field around the embedded fiber (Figure 3). This micromechanical interaction indicates the strain experienced by the FBG core differs from the strain of the surrounding composite.

Early studies examined strain transfer in embedded optical fibers. Hocker et al. [42] developed a 2D analytical model for a bare fiber under radial pressure in an isotropic host, analyzing how host material properties affect stress–strain transfer. For out-of-plane transverse loading, Mathews et al. [43] showed that a generalized plane-strain treatment is required and that equating the fiber’s axial strain to that of the host avoids errors inherent in conventional plane-strain models. For a different loading condition, Pak et al. [44] examined shear strain transfer in coated fibers under longitudinal shear loading. These studies highlight the need for precise micromechanical models to relate the sensor signal to the host strain field.

These analytical models have limitations. First, their closed-form solutions are restricted to isotropic host materials and cannot describe strain transfer in anisotropic laminates such as CFRP. Second, they are derived under idealized single-load conditions (e.g., axial tension or pure shear) and are limited to axisymmetric or simplified geometries. They lack the generality to predict complex multiaxial strain transfer in realistic composites under service loads.

To address these limitations, the analytical solution of Kollar et al. [38,45,46] for an optical fiber embedded in a transversely isotropic medium was applied here. Hence, the STP model describes the mechanical interaction via a strain transfer coefficient (STC) matrix:

(11) $$\left[\begin{array}{c} \varepsilon_{1}^{s}\\ \varepsilon_{2}^{s}\\ \vdots \\ \varepsilon_{6}^{s}\\ \Delta T^{s} \end{array}\right]=\left[\begin{array}{cccc} STC_{11} & STC_{12} & \ldots & STC_{16}\\ STC_{21} & STC_{22} & \ldots & STC_{26}\\ \vdots & \vdots & \ddots & \vdots \\ STC_{61} & STC_{62} & \ldots & STC_{66}\\ STC_{71} & STC_{72} & \ldots & STC_{76} \end{array}\begin{array}{c} STC_{17}\\ STC_{27}\\ \vdots \\ STC_{67}\\ STC_{77} \end{array}\right]\left[\begin{array}{c} \varepsilon_{1}^{H}\\ \varepsilon_{2}^{H}\\ \vdots \\ \varepsilon_{6}^{H}\\ \Delta T^{H} \end{array}\right]$$

superscripts *H* and *S* denote the far-field strain in the host and the local strain in the fiber core, respectively. Subscripts 1, 2, and 3 refer to the axial and two transverse directions. Since the FBG spectral response (Section 2.2) depends mainly on these three normal strain components and temperature (shear strain *γ*_23_ is neglected), the STC matrix simplifies to:

(12) $$\left[\begin{array}{c} \varepsilon_{1}^{s}\\ \varepsilon_{2}^{s}\\ \varepsilon_{3}^{s}\\ \Delta T^{s} \end{array}\right]=\left[\begin{array}{cccc} STC_{11} & STC_{12} & STC_{13} & STC_{14}\\ STC_{21} & STC_{22} & STC_{23} & STC_{24}\\ STC_{31} & STC_{32} & STC_{33} & STC_{34}\\ STC_{41} & STC_{42} & STC_{43} & STC_{44} \end{array}\right]\left[\begin{array}{c} \varepsilon_{1}^{H}\\ \varepsilon_{2}^{H}\\ \varepsilon_{3}^{H}\\ \Delta T^{H} \end{array}\right]$$

The STC matrix elements are determined from the analytical solution:

- Axial strain transfer is nearly complete (*STC*_11_ = 1) because the grating length is short compared to the embedded fiber, while other elements in the first row are zero;
- For thermal coupling, the fiber and host are assumed to experience the same temperature change due to intimate contact (*STC*_44_ = 1);
- Thermal expansion mismatch in the transverse directions is captured by coefficients *STC*_24_ and *STC*_34_.

The STC matrix can be computed directly from material properties of the fiber, its coating, and the host. For isotropic materials, these properties include Young’s modulus, Poisson’s ratio, and the coefficient of thermal expansion. For transversely isotropic hosts such as unidirectional CFRP laminates, the full set of elastic constants (longitudinal and transverse moduli, Poisson’s ratios, and shear moduli) are needed. This allows fast sensitivity calculation without time-consuming numerical simulations. It should be noted that the solution is based on the following assumptions: an infinitely large, transversely isotropic host, ignoring finite geometry and ply stacking effects; and a perfectly bonded interface with no damage or debonding.

## 3. Case Study and Model Verification

Based on the theoretical framework established in Section 2 for embedded FBG signal response under multiaxial strain transfer, systematic validations were carried out. The responses of the embedded FBG under three multiaxial loading conditions including hydrostatic pressure [22], transverse biaxial compression [21], and biaxial tension [39] were calculated with the theoretical framework and were examined with the measurement data.

The hydrostatic and biaxial compression tests assess the predictive accuracy and computational efficiency of the STP theory under ideal interface conditions. The biaxial tension tests, in contrast, examine the analytical model’s limitations in non-ideal scenarios such as interfacial debonding.

Through this comparative validation, the relationship between FBG spectral signals and the host’s local strain field were discussed to reveal the signal misinterpretation under complex service loads.

### 3.1. Influence of Hydrostatic Pressure

The hydrostatic pressure test data used for validation are taken from the experimental study by Lai et al. [22]. In their work, an uncoated FBG was embedded in both an epoxy resin specimen and a unidirectional CFRP laminate, and the Bragg wavelength shift was recorded under hydrostatic loading. Because hydrostatic pressure produces uniform compression in all directions, the embedded FBG experiences negligible birefringence, resulting in a symmetric reflection spectrum without broadening or splitting.

The epoxy specimen was a solid cylinder (radius: 12 mm, length: 40 mm) with the FBG embedded along its central axis at the longitudinal midpoint. The CFRP specimen measured 36 mm × 28 mm × 4.2 mm, fabricated by autoclave curing with a [0]_24_ stacking sequence, and the FBG was embedded in the mid-ply, aligned with the fiber direction at the geometric center.

While Lai et al. [22] provided both experimental data and a numerical analysis of axial strain sensitivity, they did not detail the strain transfer mechanism between the host and the embedded fiber across different materials. To address this gap, we adopted a comparative finite element (FE) modelling approach using two models: one with the embedded FBG and one without. Comparing the FE results directly quantifies the local strain field perturbation caused by the FBG. The strain field from the no-FBG model is then fed into the embedded FBG signal response theory developed in Section 2 to predict the spectral response.

**Figure 4:**
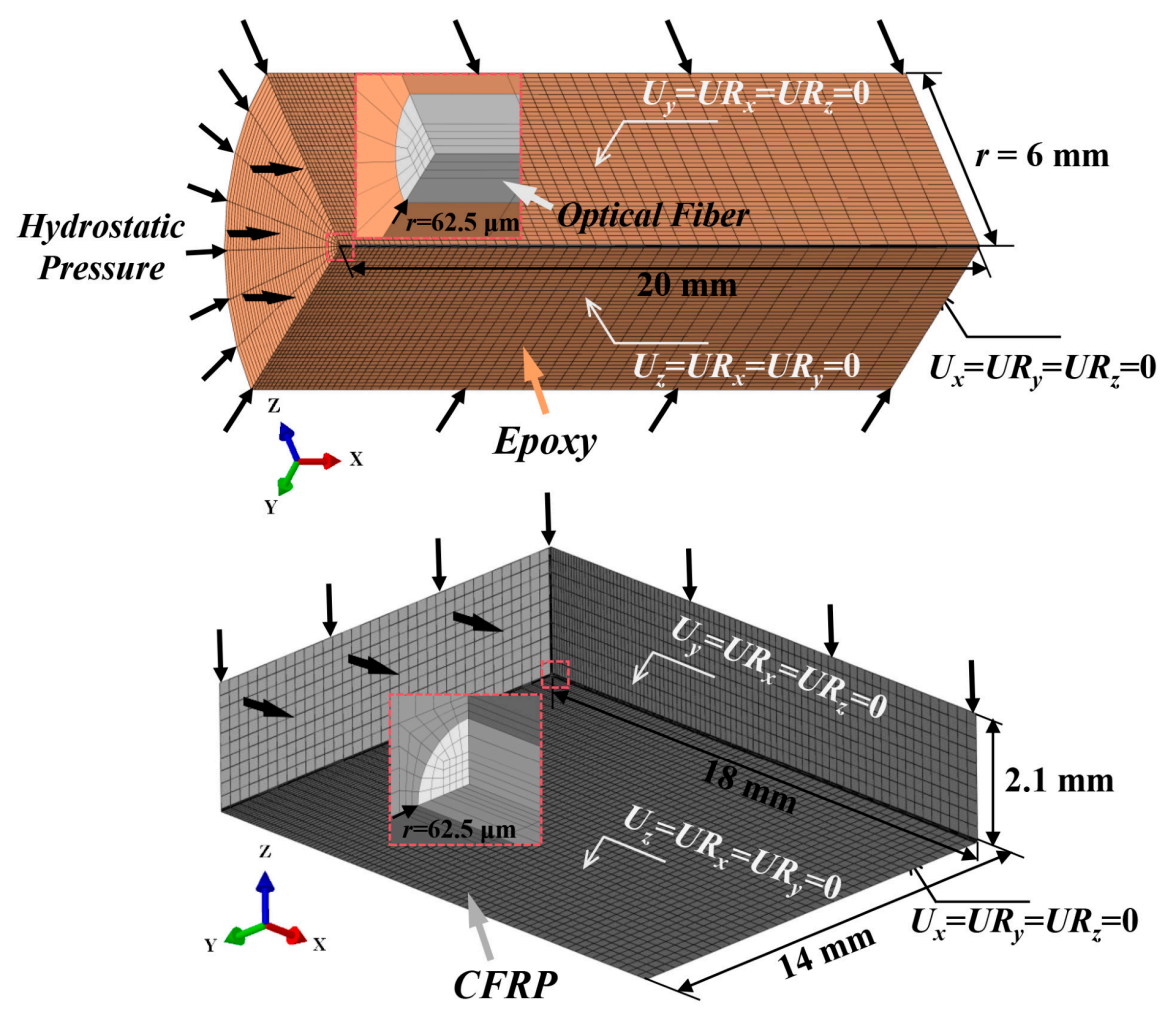
Schematic diagram of hydrostatic pressure numerical model.

The numerical models followed the experimental geometry and loading conditions, as shown in Figure 4. Owing to symmetry in geometry, materials, and loading, both the epoxy cylinder and the CFRP laminate were modelled using a one-eighth symmetry scheme, with appropriate symmetric boundary conditions applied on the planes. The material properties for both the epoxy and CFRP are taken from Lai et al. [22] and are summarized in Table 1. A uniform hydrostatic pressure was applied to the outer surfaces at magnitudes equal to the experimental maxima: 9.2 MPa for epoxy and 13 MPa for CFRP.A mesh sensitivity analysis was conducted to ensure that the element sizes around the fiber interface do not affect the predicted strain fields. The same mesh sensitivity analysis was conducted for the subsequent biaxial compression models.

#### 3.1.1. Host Material: Epoxy

**Figure 5:**
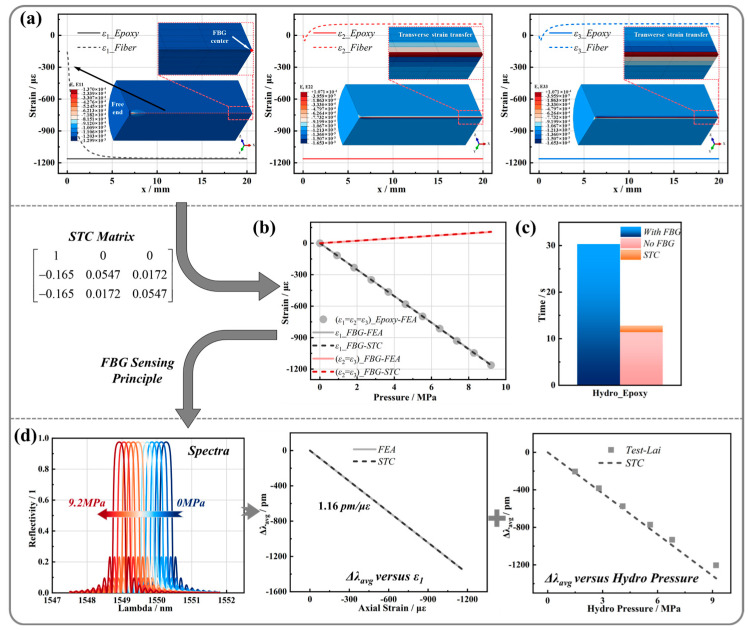
Embedded FBG signals under epoxy hydrostatic loading: (**a**) strain distribution along the specimen axis at 9.2 MPa; (**b**) strain components and (**c**) computing time between STP and numerical simulation; (**d**) embedded FBG signals.

Under 9.2 MPa hydrostatic pressure, the pure epoxy model shows a uniform and isotropic strain state. The model with an embedded FBG, however, exhibits localized perturbations as shown in Figure 5a. Axial strain concentrates at the fiber entrance but stabilizes within about 5 mm, after which its radial gradient vanishes. Within this steady region, *ε*_1_ in the FBG equals that of the host epoxy. This confirms the axial strain transfer coefficient of unity (*STC*_11_ = 1) assumed in the analytical STC matrix.

Transverse strains *ε*_2_ and *ε*_3_ also stabilize quickly but exhibit pronounced transverse gradients near the host-fiber interface. This indicates incomplete transverse strain transfer from the epoxy to the FBG since the transverse strains neither match those of the epoxy nor follow a simple Poisson relation with the axial strain. This indicates a more complex multiaxial strain coupling mechanism.

The STC matrix derived from epoxy material parameters is shown in Figure 5. Analysis of this matrix shows that the transverse strain components at the FBG sensing point are coupled with all three principal strains of the host. Moreover, axial strain in the host exerts a dominant and equal negative influence on the transverse strains at the sensing point, reflected in the coefficients *STC*_21_ = *STC*_31_ = −0.165. The submatrix formed by the second and third rows and columns is symmetric, indicating consistent transverse coupling.

Comparing the FBG center strain from STP-based prediction against the FBG-included numerical simulation shows excellent agreement, with minimal errors in all three components as shown in Figure 5b. This confirms that the STC is an alternative to detailed FBG modeling for predicting strain under hydrostatic loading in isotropic media. The comparison also shows the method’s computational efficiency, reducing the cost of detailed FBG-embedded simulations (Figure 5c).

The strain field from the STC matrix agrees with direct numerical simulation at the FBG location. This confirms that the STP method predicts the FBG spectral response under hydrostatic pressure. As shown in Figure 5d, the transverse strain difference remains stable during loading, causing no spectral broadening or distortion. From the acquired spectra, we extracted the average spectral shift Δ*λ_avg_* and plotted it against both the axial mechanical strain *ε_1_* and the hydrostatic pressure. The apparent axial strain sensitivity is defined as the ratio of the average spectral shift Δ*λ_avg_* to the axial mechanical strain in the grating region:

(13) $$K_{\varepsilon }^{avg}=\frac{\Delta \lambda_{avg}}{\Delta \varepsilon_{1,m}}$$

The STP-based method gives a value of 1.16 pm/με, which matches the simulation result (Figure 5d). The average spectral shift predicted by the STP theory as a function of pressure differs from the experimental measurement by 7.6%. This discrepancy is mainly due to uncertainties in the measured epoxy mechanical properties and experimental noise. The small error confirms that the host-fiber interface remained bonded throughout the experiment.

The calculated apparent axial sensitivity *K_ε_^avg^* under hydrostatic pressure is about 4.1% lower than the theoretical *K_ε_* value of 1.21 pm/με, which is calculated from the FBG parameters provided by Lai et al. for a bare FBG under the axial-stress assumption. This suggests that directly using the *K_ε_* to derive axial strain from wavelength shift introduces an acceptable error for epoxy under hydrostatic loading. However, this simplification does not hold when the host is a composite, as discussed later.

#### 3.1.2. Host Material: CFRP

**Figure 6:**
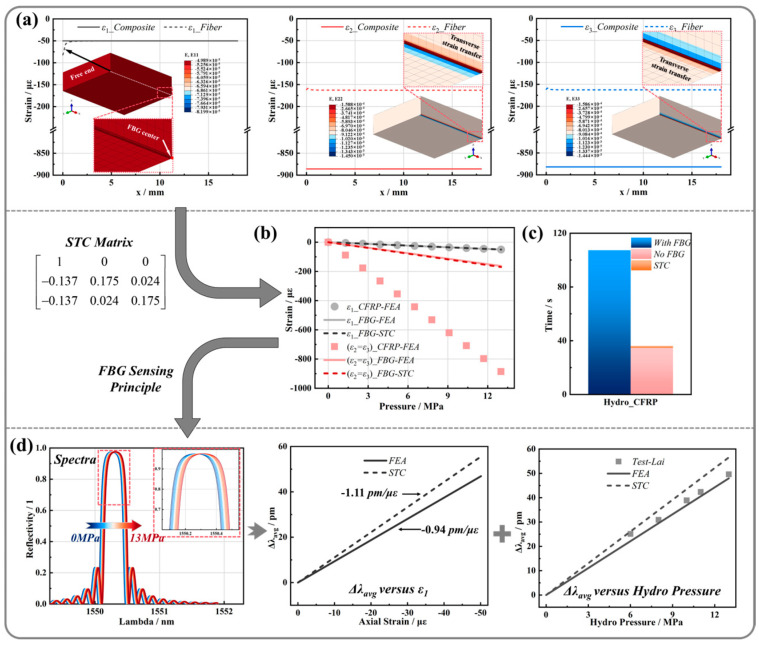
Embedded FBG signals under CFRP hydrostatic loading: (**a**) strain distribution along the specimen axis at 13 MPa; (**b**) strain components and (**c**) computing time between STP and numerical simulation; (**d**) embedded FBG signals.

Under 13 MPa hydrostatic pressure, the embedded FBG in CFRP induces a localized strain perturbation as shown in Figure 6a.

Compared with the epoxy host, the strain disturbance near the fiber entrance is narrower in CFRP. Within the stabilized grating region, the axial strain in the FBG equals that of the CFRP host, confirming complete axial strain transfer as the epoxy case. As observed in epoxy, the transverse strain in the FBG neither matches the matrix transverse strain nor follows the Poisson relationship with the axial strain.

The grating length allows the host axial strain to be fully transmitted to the grating region through interfacial shear. Complete axial strain transfer (*STC*_11_ = 1) occurs in both epoxy and CFRP. The host material’s elastic modulus affects only the length of the end region where axial strain transfer is incomplete. It does not influence the full transfer within the grating region.

In the transverse directions, the elastic properties of the FBG and host are mismatched. The host modulus (1.9 GPa for epoxy, 8.7 GPa in the transverse direction of CFRP) is much lower than the FBG modulus of 72 GPa. Their Poisson ratios also differ. This mismatch prevents the FBG from deforming with the host under transverse loading. The constraint from the surrounding host restricts the FBG from deforming through the Poisson effect from axial strain alone. Compounding this incompatibility, the FBG radius is only 125 μm, orders of magnitude smaller than the host dimensions. This size disparity hinders transverse strain transfer, as the FBG’s small cross-section makes it susceptible to the property mismatch.

Following the same methodology, the STC matrix for CFRP was derived from its material parameters (Figure 6). The axial strain of the host exerts an equal negative influence on the transverse strains, reflected in the coefficients *STC*_21_ = *STC*_31_ = −0.137. However, unlike in isotropic epoxy, the host axial strain is no longer the dominant coupling term. This results from the high axial stiffness of CFRP relative to its transverse stiffness. With an axial modulus of 96 GPa and transverse modulus of 8.7 GPa, the material limits Poisson-induced transverse deformation. The contribution of axial strain to the transverse strains of the FBG is reduced. Instead, the transverse strains of the FBG are governed by those of the host. This comes from the higher transverse modulus of CFRP compared to epoxy. This allows more transmission of transverse deformation from the host to the FBG, reflected in the larger coefficients *STC*_22_ = *STC*_33_ = 0.175.

The comparison between strains from the FBG-embedded simulation and the STP-based prediction shows good agreement in Figure 6b. The axial strains are nearly identical, while discrepancies in the transverse strains are minimal. This match demonstrates that the STC matrix is not limited to isotropic media like epoxy but also applies to transversely isotropic materials such as CFRP.

As shown in Figure 6d, the *K_ε_^avg^* for CFRP under hydrostatic pressure was evaluated. The FBG-included FE simulation gives −0.94 pm/με. The STP-based model predicts −1.11 pm/με. Both values differ in magnitude and sign from the theoretical *K_ε_* of 1.21 pm/με under the axial stress assumption. This change comes from the high axial stiffness of CFRP, which constrains axial deformation of the embedded FBG. The spectra did not distort during loading, indicating that even when the FBG reflection spectrum maintains a stable shape under complex loading, the axial strain sensitivity must be recalibrated for the CFRP host. Both the detailed numerical simulation and the STP model agree with the experimental data, with discrepancies of 5.3% and 10.6%, respectively (Figure 6d). This confirms that the STP method maintains accuracy while being computationally efficient (Figure 6c).

Under hydrostatic pressure, applying the *K_ε_* of 1.21 pm/με without recalibration would produce different strain errors for the two host materials. For epoxy, the error would be within 5%. For CFRP, however, the strain magnitude is overestimated by more than 200%, and the sign would be inverted which led to dramatical misinterpreting compression as tension.

### 3.2. Influence of Biaxial Compression Loading

The biaxial compression test data were sourced from Bosia et al. [21], in which an FBG was embedded at the center of an epoxy cube (30 × 30 × 40 mm^3^) and subjected to two symmetric loading configurations. Reflected signals from the two orthogonal polarization modes were recorded to characterize the fast- and slow-axis responses. Although Bosia et al. [21] developed a numerical model to study wavelength shifts and peak splitting in epoxy under biaxial compression, their analysis was confined to this isotropic system and did not yield an STC matrix for multiaxial strain transfer analysis.

**Figure 7:**
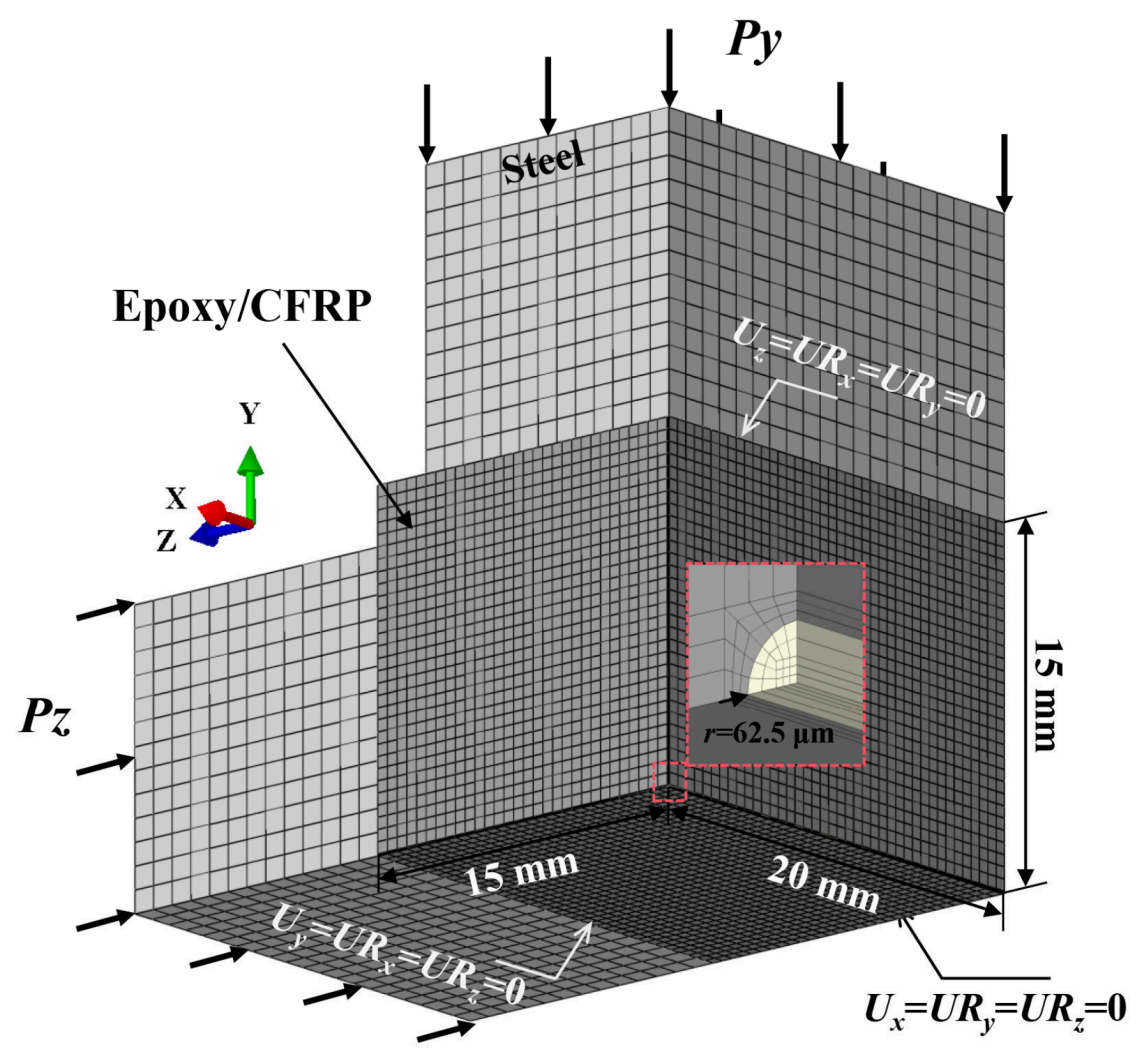
Schematic diagram of biaxial compression numerical model.

To bridge this gap, two FE models (with and without the FBG) were created based on the experimental setup. Exploiting geometric and loading symmetry, a one-eighth symmetry model with appropriate boundary conditions was adopted (Figure 7). The simulation applied a fixed horizontal load *Py* = 1 kN while varying the vertical load *Pz* from 1 to 45 kN, consistent with the experimental loading scheme, and incorporated the steel compression fixtures (E = 207 GPa, ν = 0.29), with a friction coefficient of 0.2 at the fixture–specimen interface. For the CFRP configuration, the host material was modelled as a unidirectional laminate with the FBG embedded parallel to the fiber direction. The specimen dimensions and FBG placement remained the same as in the epoxy case, with biaxial compression applied in the two transverse directions.

The analysis proceeded in two steps. First, the strain at the FBG location predicted by the STC matrix was compared with the direct results from the detailed model (with fiber) to validate the STP accuracy. Subsequently, the validated strain field was processed through the opto-mechanical model to generate spectral predictions. Material properties for epoxy were taken from Bosia et al. [21], and those for CFRP from Lai et al. [22].

#### 3.2.1. Host Material: Epoxy

**Figure 8:**
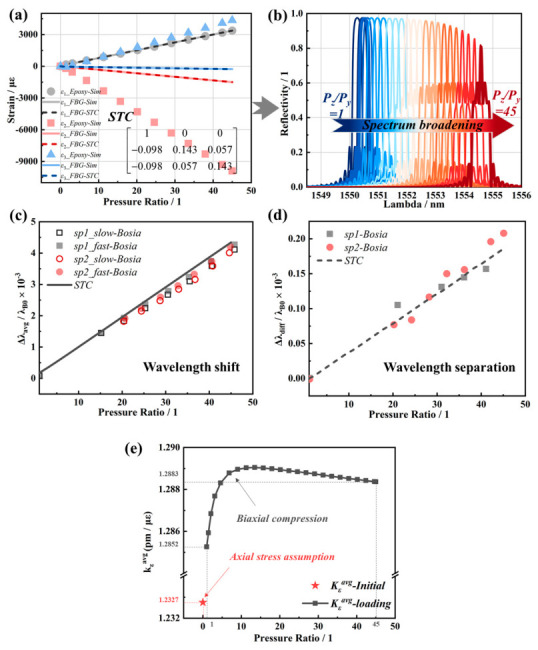
Strain transfer of embedded FBG in epoxy during biaxial compression: (**a**) strain components as functions of load ratio; (**b**) spectra; (**c**) wavelength shift; (**d**) wavelength separation; (**e**) apparent axial strain sensitivity coefficient.

First, the STC matrices are compared for two epoxy systems with different stiffnesses: one used in hydrostatic pressure (Figure 5, E = 1.9 GPa) and the other in biaxial compression (Figure 8a, E = 2.5 GPa). Both have a Poisson’s ratio near 0.4. The increase in host stiffness shifts the dominant coupling toward FBG’s transverse strain. In the softer resin, the host’s axial strain exerts a stronger influence (e.g., *STC*_21_ = −0.165 vs. *STC*_22_ = 0.0547). The relationship reverses in the stiffer resin, and the host’s transverse strain has a more significant effect (e.g., *STC*_21_ = −0.098 vs. *STC*_22_ = 0.143). This trend occurs because the host’s transverse strain increasingly dominates the FBG’s transverse response as the host stiffness increases. In the theoretical limit where the host modulus matches that of the FBG’s silica glass, perfect strain transfer would occur, yielding *STC*_21_ = *STC*_23_ = 0 and *STC*_22_ = 1.

Figure 8a also plots the evolution of strain components at the FBG versus the pressure ratio. The STC-predicted strains show agreement with the detailed FE results over the entire loading range. Unlike under hydrostatic pressure, the transverse strain difference |*ε*_2_ − *ε*_3_| increases with the biaxial compression pressure ratio. This occurs because the in-plane transverse pressure on the epoxy host is held constant while the out-of-plane transverse pressure increases, creating a disparity in the transverse strains transferred to the grating region. The resulting strain asymmetry induces a birefringence effect, which broadening the FBG spectra gradually accompanied by a reduction in peak intensity, as shown in Figure 8b.

As shown in Figure 8c,d, the STC-predicted Bragg wavelength shifts and the separation between the fast and slow axes both show agreement with experimental measurements. This confirms the reliability of the present framework under biaxial compression. It also validates the assumption of the STP that the interfacial bonding between the FBG sensor and the epoxy host remains intact. This assumption, however, does not hold under all loading conditions, as shown in Section 3.3 on biaxial tension.

Figure 8e shows the apparent axial strain sensitivity coefficient *K_ε_^avg^* of the embedded FBG versus the biaxial compression load ratio (*P_z_*/*P_y_*), with the coefficient calculated between the unloaded initial state and each loaded state. Because both loading and structure are symmetric, the response for load ratios in (0, 1] mirrors that for ratios in [1, +∞) on a logarithmic scale symmetric about *P_z_*/*P_y_* = 1. Only ratios ≥1 are shown.

Two observations are found. First, under any load ratio, the coefficient deviates from the theoretical *K_ε_* of 1.2327 pm/με (calculated for a bare FBG under the pure axial stress assumption using the fiber parameters reported by Bosia et al. [21])) due to the non-axial stress state at the grating under biaxial compression. Second, although the axial strain and Bragg wavelength shift at the FBG increase nearly linearly with load ratio (Figure 8a,c), *K_ε_^avg^* varies nonlinearly. It rises from 1.2852 pm/με at the 1:1 load ratio and goes to 1.2883 pm/με. This nonlinear trend results from a shift in the transverse stress state at the grating: as the out-of-plane compression increases, its influence gradually dominates over the constant in-plane compression, modifying the strain field transferred from the host.

Despite this nonlinearity, the variation range of the coefficient is narrow (1.285–1.289 pm/με). The stabilized value of 1.288 pm/με can represent *K_ε_^avg^* for this loading condition. This value differs from the theoretical *K_ε_* by 4.3%, indicating that the error introduced by directly applying the axial value in measurement is acceptable.

#### 3.2.2. Host Material: CFRP

The analysis considers unidirectional laminates with an FBG aligned parallel to the embedded FBG axis and subjected to biaxial compression in two transverse directions. Experimental validation for this CFRP case was not conducted.

**Figure 9:**
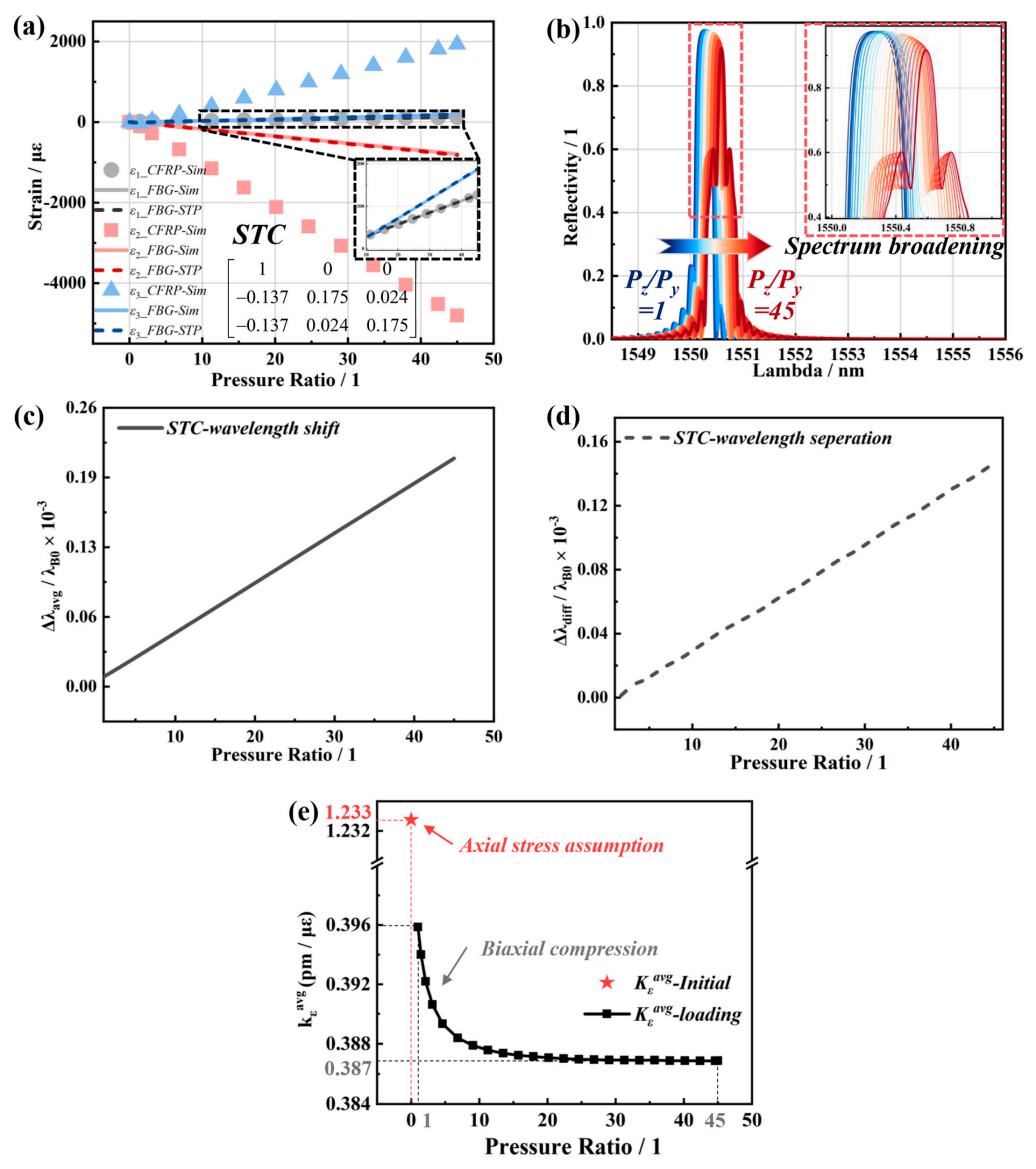
Strain transfer of embedded FBG in CFRP during biaxial compression: (**a**) strain components as functions of load ratio; (**b**) spectra; (**c**) wavelength shift; (**d**) wavelength separation; (**e**) apparent axial strain sensitivity coefficient.

Figure 9a compares the FBG strains predicted by the STC matrix with those from the fiber-included finite element model. The agreement over the entire loading range validates the STP-based framework for CFRP under biaxial compression.

The transverse strain difference |*ε*_2_ − *ε*_3_| at the FBG increases with the load ratio, causing the spectral broadening seen in Figure 9b. The quantitative degree of broadening (black dashed line in Figure 9d) is slightly lower than that in the epoxy case in Figure 9d. The transverse modulus of the unidirectional carbon fiber reinforced plastic laminates is at the same order of magnitude as that of the epoxy resin, and accordingly, the transverse strain transfer coefficients are comparable (*STC*_22_ = 0.143 for epoxy vs. 0.175 for CFRP). Thus, the two material systems show similar spectral broadening behavior.

Due to the high modulus along fiber direction, the longitudinal strain change under biaxial compression is small, about 100 με (Figure 9a). The overall center wavelength shift of the embedded FBG is much smaller than that in epoxy. A smaller redshift of the spectrum is observed in Figure 9b and is quantified in Figure 9c (black solid line). This behavior is reflected in the *K_ε_^avg^* for CFRP-embedded FBGs under biaxial compression, as shown in Figure 9e. The coefficient decreases from 0.396 pm/με at *P_z_*/*P_y_* = 1 and stabilizes at 0.387 pm/με, a value lower than the theoretical *K_ε_* of 1.2327 pm/με derived under the axial stress assumption. It is notable that using the theoretical *K_ε_* to convert the measured wavelength shift into host axial strain would introduce an error of about 70%.

### 3.3. Influence of Biaxial Tension Loading

The hydrostatic pressure and biaxial compression results demonstrate that the STP-based framework reliably predicts embedded FBG responses and provides accurate case-specific *K_ε_^avg^* values for both epoxy and CFRP hosts. This approach is effective for converting measured wavelength shifts into axial strain at the monitoring location under a perfectly bonded FBG–host interface. However, this ideal condition is not always maintained, as reported in the biaxial tension experiments of Martínez et al. [39], where the FBG wavelength shift predicted from strain-gauge measurements and strain-optic theory exhibited the opposite trend to the experimental observation. They attributed this discrepancy to incomplete transverse strain transfer in CFRP but did not provide validation through FE or analytical methods. To address this, an investigation of imperfect bonding effects was conducted here using the same STP-based framework.

In the experiments of Martínez et al. [39], CFRP specimens with a [90/0]_5s_ stacking sequence were tested under biaxial tension, with the FBG embedded at the mid-plane and aligned with the 0° fiber direction. The longitudinal strain (along the 0° fiber direction) was held at several fixed levels, while the in-plane transverse strain was incrementally increased and decreased along both loading and unloading paths. A strain gauge rosette mounted on the specimen surface provided real-time measurements of the axial and transverse strains at the location corresponding to the embedded FBG.

**Figure 10:**
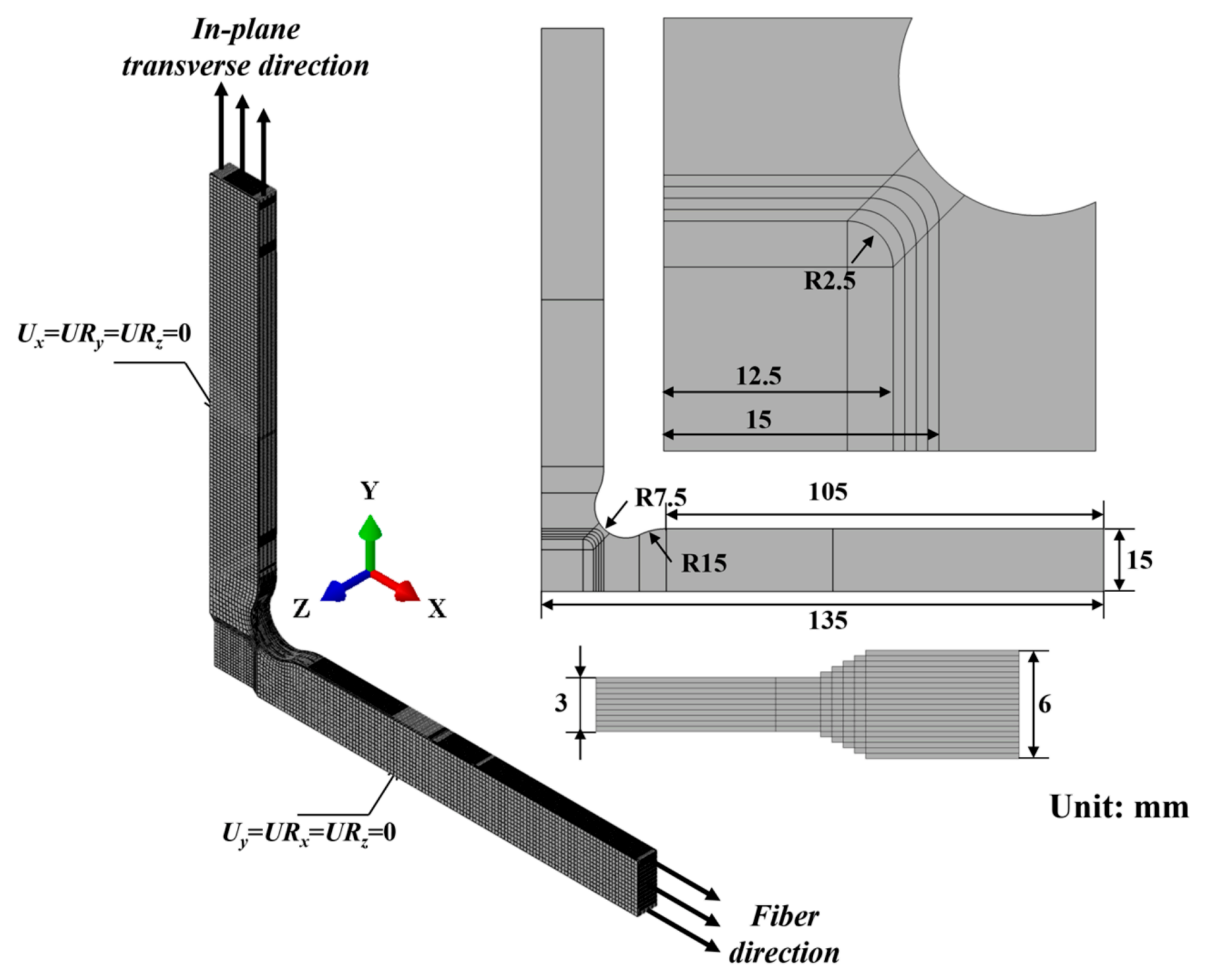
Schematic diagram of biaxial tension numerical model.

In this study, a quarter-symmetry numerical model was created following the experimental specimen geometry (Figure 10). To reduce computational cost, the embedded FBG was omitted. The pure CFRP laminate was first simulated under biaxial tension to obtain its macroscopic strain field. The simulation followed the same loading protocol as the experiments: the longitudinal strain was held at fixed levels of 500, 1000, 1500, and 2000 με (cases 1–4), while the in-plane transverse strain was incrementally varied from 0 to 4000 με. The strain field was then transferred to the FBG via the STC matrix to calculate the spectral response, enabling an assessment of the imperfect bonding effect.

#### 3.3.1. Simulation Analysis

**Figure 11:**
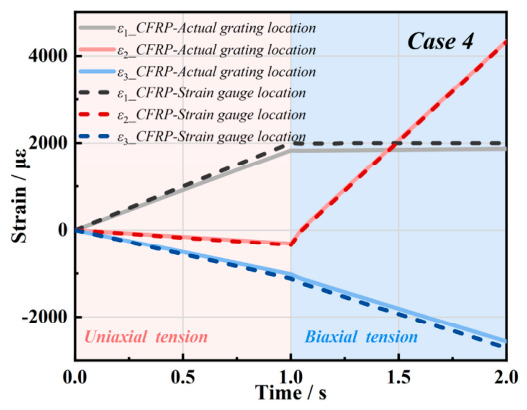
Comparison of strain fields at the strain rosette and embedded FBG locations for biaxial tension case 4.

In experiments, the strain levels for cases 1–4 were controlled using a surface-mounted strain rosette at the specimen center. However, as shown in the uniaxial tension simulation (Figures S1–S3 in the Supplementary Material), the actual embedded FBG was not at the specimen center [47]. To account for this discrepancy, the surface strain at the specimen center was used as the loading indicator. The local CFRP strain field at the actual FBG embedding location was extracted for subsequent FBG signal analysis. Figure 11 presents the CFRP strains at these two locations for case 4, revealing a discrepancy. Similar discrepancies were also observed for cases 1–3.

The loading procedure consisted of two stages: the axial strain was first increased to 2000 με and held constant, after which the in-plane transverse strain was applied.

**Figure 12:**
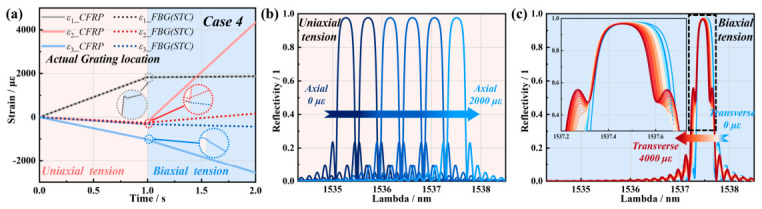
Strain transfer characteristics of the embedded FBG in CFRP for biaxial tension case 4: (**a**) evolution of strain components with time; (**b**) FBG spectra during uniaxial tension; (**c**) FBG spectra during biaxial tension.

During the transition from uniaxial to biaxial loading, two features were observed in the strain field at the FBG location (Figure 12a). First, a small perturbation occurred at the transition point where transverse tension was applied while maintaining constant axial strain. Second, after the transition, the axial strain at the actual FBG position did not stay constant at 2000 με. Because of the positional misalignment between the FBG and the specimen center, it increased linearly to about 1900 με.

Applying the STC matrix to the CFRP strain field at the embedding location gives the strain field of the FBG. From this, the spectral response of the embedded FBG was determined for the uniaxial tension stage and the subsequent biaxial tension stage (Figure 12b,c). During the uniaxial tension stage, the spectra showed a pure positive shift without broadening. In the biaxial tension stage, the axial strain remained nearly constant, causing a small negative shift in the spectrum. The in-plane transverse tension increased the transverse strain difference, which led to continuous spectral broadening (Figure 12c).

**Figure 13:**
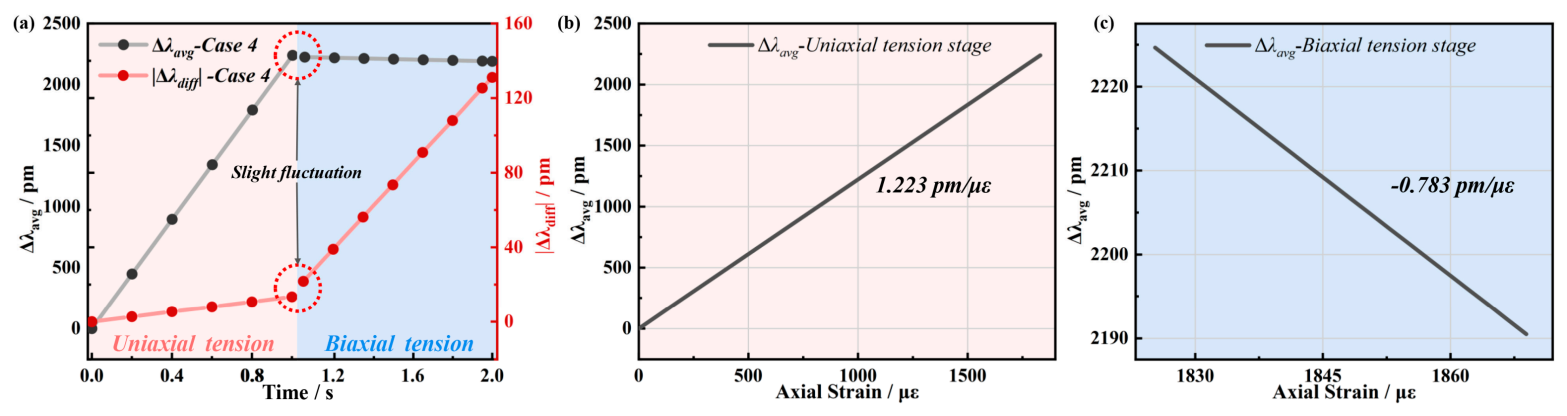
Embedded FBG response for biaxial tension case 4: (**a**) wavelength shift and separation; (**b**) *K_ε_^avg^* during uniaxial tension; (**c**) *K_ε_^avg^* during biaxial tension.

The wavelength shift and separation were extracted over the entire loading process for case 4, as shown in Figure 13a. During both the uniaxial and biaxial tension stages, these two parameters evolved linearly with time. A slight fluctuation was observed at the transition between the two stress states, highlighted by the dashed circle in Figure 13a. This fluctuation originated from the strain-field perturbation (Figure 12a) caused by transient adjustments in the simulation setup. It did not affect the analysis of the subsequent linear trends.

Once the strain field stabilized, the linear evolution resumed in biaxial tension stage. The average wavelength shift Δ*λ_avg_* was plotted against the axial strain separately for the uniaxial and biaxial tension stages. The resulting *K_ε_^avg^* values were 1.223 pm/με for uniaxial tension and −0.783 pm/με for biaxial tension, as shown in Figure 13b,c.

Table 2 presents the wavelength shift and spectral separation for cases 1–4 during both the uniaxial and biaxial tension stages. It is calculated using the STP-based framework proposed in this study. At the beginning of each respective loading stage, both Δ*λ_avg_* and |Δ*λ_diff_*| were referenced to zero.

During the uniaxial tension stage, Δ*λ_avg_* increased linearly with the applied axial strain, reaching values between 560 pm and 2239 pm for cases 1–4. As expected, the axial strain sensitivity *K_ε_^avg^* of the embedded FBG remained constant at 1.223 pm/με, since the uniaxial tension condition imposed an ideal axial stress state on the FBG. The spectral separation |Δ*λ_diff_*| stayed small throughout this stage, ranging from 3.4 pm to 13.4 pm.

During the biaxial tension stage, Δ*λ_avg_* became negative and stayed within a narrow range of −31.6 to −37.6 pm across all cases. According to Equation (10), Δ*λ_avg_* was governed by both Δ*ε*_1_*^S^* and (Δ*ε*_2_*^S^*+ Δ*ε*_3_*^S^*). In this stage, the in-plane transverse tension load was progressively increased, while the axial load was adjusted to keep the axial strain constant at the strain rosette location. Because of the positional misalignment between the FBG embedded location at (5.4, 9, 3) and originally designed location at (0, 0, 3) (Figure S1), Δ*ε*_1_*^S^* at the FBG location increased to a similar level (approximately 40 με) in all cases. At the same time, (Δ*ε*_2_ + Δ*ε*_3_) induced by the transverse tension load also reached comparable magnitudes across the different loading conditions. Substituting these two factors into Equation (10) gave a negative component to Δ*λ_avg_*.

The variation in Δ*λ_avg_* across the four cases was primarily caused by the positional discrepancy mentioned earlier, leading to fluctuations in the calculated *K_ε_^avg^*.

(14) $$\frac{\Delta \lambda_{avg}}{\lambda_{B}}=-\frac{n_{eff,0}^{2}}{2}\left[\frac{p_{11}+p_{12}}{2}\left(STC_{22}+STC_{23}\right)\left(\Delta \varepsilon_{2}^{H}+\Delta \varepsilon_{3}^{H}\right)\right]$$

If the FBG had been embedded exactly at the specimen center as intended, the axial strain variation of the host at the specimen center, Δ*ε*_1_*^H^*, would be zero during biaxial tension stage. Using the STC matrix, Δ*λ_avg_* can then be computed from Equation (14). In this case, with Δ*ε*_1_*^H^* = 0 and an applied transverse strain Δ*ε*_2_*^H^* = 4000 με in the CFRP under plane stress and zero shear, Δ*ε*_3_*^H^* arises solely from the Poisson effect and is determined using Classical Laminate Theory (CLT). The differences in Δ*ε*_3_*^H^* across the four cases are only tens of micro strain, so (Δ*ε*_2_*^H^* + Δ*ε*_3_*^H^*) remains nearly the same for each case. It would yield almost indistinguishable Δ*λ_avg_* values across all cases.

If the axial strain variation Δ*ε*_1_ used to compute *K_ε_^avg^* were taken from the surface strain rosette location rather than the actual FBG embedding position, *K_ε_^avg^* could not be defined from the slope of Δ*λ_avg_* versus Δ*ε*_1_. This is because the axial strain at the rosette location remains constant during the biaxial stage by design, resulting in no strain variation.

During the biaxial tension stage, the spectral separation |Δ*λ_diff_*| increased to 115.6–117.6 pm, nearly identical across all four cases despite different initial axial strain magnitudes. This consistency arises because |Δ*λ_diff_*| is governed by the transverse strain difference (Δ*ε*_2_*^S^* − Δ*ε*_3_*^S^*) (Equation (10)), and this difference remained unaffected by the positional misalignment of the embedded FBG.

Although the positional misalignment caused variations in Δ*λ_avg_* across the four cases, the following key conclusions remain valid. First, during the biaxial tension stage, Δ*λ_avg_* consistently exhibited a small negative shift of approximately −35 pm. Second, this shift was independent of the initial axial strain magnitude.

#### 3.3.2. Experiment Validation

**Figure 14:**
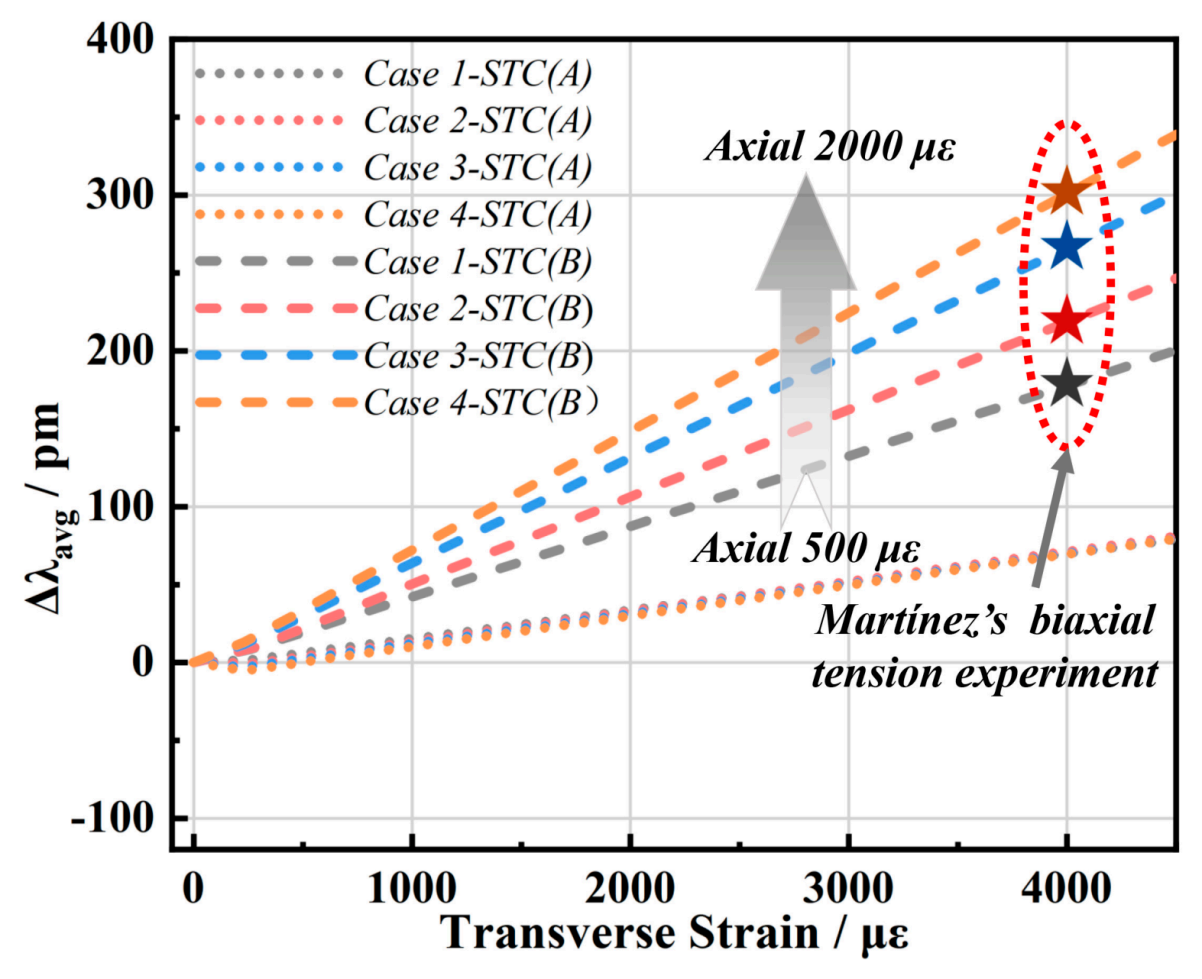
Comparison of experimental and modified STC matrix predicted wavelength shifts of the embedded FBG during biaxial tension.

However, experimental results from Martínez et al. [39] show that the initial axial strain level influences the FBG response, as shown in Figure 14. For a fixed longitudinal strain, the wavelength shift increases with applied transverse strain. At a transverse strain of 4000 με, the measured wavelength shifts reach 179, 219, 266, and 301 pm for fixed axial strains of 500, 1000, 1500, and 2000 με, respectively.

In contrast, the numerical analysis in the previous subsection shows that during biaxial tension, Δ*λ_avg_* exhibits a small, nearly constant negative shift (about −35 pm) independent of the initial axial strain magnitude. This analysis is based on the STC matrix, which assumes perfect interfacial bonding between the host material and the FBG. However, the finding contrasts with the experimental observations, indicating that the realistic condition may be beyond the ideal strain transfer mechanism.

**Figure 15:**
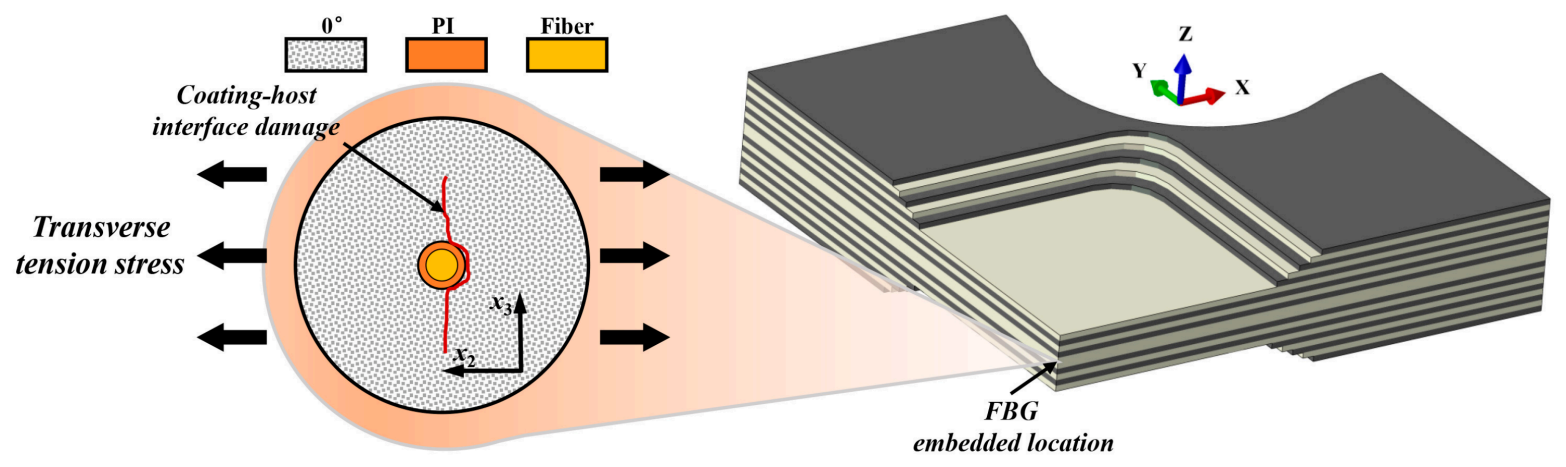
Coating-host interface damage.

A plausible explanation is progressive evolution of interfacial damage under increasing transverse tension. Previous studies have shown that the interface between embedded FBGs and the composite host is highly prone to damage under transverse tension [37] or residual curing stresses [48]. Such damage can significantly alter the strain transfer between the host material and the FBG and ultimately change the sensing response of the embedded FBG. A schematic illustration of this interfacial damage is shown in Figure 15.

To enable a semi-quantitative analysis of how such interfacial damage affects the response of the embedded FBG, Figure 14 compares the experimental wavelength shifts with predictions from a modified STC matrix. In the figure, the predicted wavelength shifts are plotted, with star symbols marking the experimental values at a transverse strain of 4000 με for each case. The STC matrix coefficients used in the predictions are listed in Table 3, which includes two modified sets (Entries A and B) representing different interfacial damage conditions. These matrices are adapted from the original STC matrix that assumes perfect interfacial bonding.

The STC matrix characterizes the strain transfer between the host and the sensor, with its original parameters derived under perfect bonding. This original formulation, however, failed to capture the experimental trends under biaxial tension.

To investigate the influence of interfacial damage, a modification was introduced by setting *STC*_22_ = 0 (entry A in Table 3). As the parameter *STC*_22_ governing in-plane transverse strain transfer, setting it to zero represents a scenario where such strain cannot be transmitted to the FBG due to interfacial damage. After this adjustment, the correlation between wavelength shift and transverse strain changed from negative to positive, aligning with the experimental trend indicated by the dotted line in Figure 14. Nevertheless, the modified model cannot fully reproduce the experimental observation that, at a given transverse strain, the wavelength shift increases with the initial axial strain.

B cases in Table 3 accounts for through-thickness interfacial cracks that hinder strain transfer from the host to the FBG in the 2-direction. Accordingly, *STC*_22_ = *STC*_32_ = 0 are assumed to represent a fully damaged transverse interface. The emergence of such cracks alters the local constraint state of the FBG under biaxial tension. When axial strain develops along the fiber direction, the constraint in the 3-direction remains intact, whereas that in the 2-direction is partially relaxed. Therefore, *STC*_31_ is kept unchanged, while *STC*_21_ is reduced to half of its original value. The remaining coefficients, *STC*_23_ and *STC*_33_, are adjusted to ensure that the simulated central wavelength shift of the embedded FBG remains consistent with experimental observations while maintaining a non-broadened spectrum. Based on these assumptions, the obtained parameters *STC*_23_ and *STC*_33_ increase approximately linearly with axial strain. For cases 1–4, both coefficients take the same values of 0.178, 0.228, 0.286, and 0.328, respectively.

This B-modified approach yields predictions consistent with the experimental findings of Martínez et al. [39]. As shown by the short-dashed line in Figure 14, the predicted Δ*λ_avg_* during transverse tension increases with the initial axial strain level, a trend that aligns with the experimental values (star symbols) reported in their study.

These results confirm that interfacial damage under coupled axial–transverse loading alters transverse strain transfer. To identify how the interfacial crack initiates and evolves, in situ X-ray CT characterization and refined numerical modeling should be carried out further. Such an investigation is beyond the scope of this study.

## 4. Conclusions

This study clarifies the relationship between embedded FBG’s response with the host’s local strain field under multiaxial loading. Systematic investigations were carried out under three representative loading conditions including hydrostatic pressure, biaxial compression, and biaxial tension in epoxy and CFRP host materials.

The proposed STP-based framework analytically calculates strain transfer from constituent material properties and was validated through hydrostatic and biaxial compression tests, yielding prediction errors below 5%. It is shown that the bare FBG coefficient *K_ε_* is not applicable for multiaxial loadings such as hydrostatic pressure and biaxial compression. For hydrostatic pressure cases, the apparent coefficient *K_ε_^avg^* reaches about −1.11 pm/με, which can not only cause large amplitude errors but also invert the sign of the interpreted strain, indicating that the sensitivity coefficient must be recalibrated for the real composite host and loading condition. Biaxial compression results further reveal that the spectral separation between the fast and slow axes captures variations in the host’s transverse strain difference, underscoring the critical importance of interrogation systems that support both central wavelength tracking and real-time extraction of spectral width and separation.

Under biaxial tension, in-plane transverse tensile stress can initiate FBG-host interfacial damage that fundamentally alters transverse strain transfer. The STP method based on ideal interfacial bonding cannot capture progressive interfacial damage or its effect on signal interpretation under realistic conditions, which requires further investigation.

These findings fill a gap in understanding the embedded FBG response under multiaxial loading and provide a systematic theoretical basis for embedded FBG SHM applications in composite materials.

## Figures and Tables

**Table 1:** Hydrostatic Pressure Material Parameters [22].

Parameters	FBG	Parameters	Epoxy	Parameters	CFRP
*E*	72 GPa	*E*	1.9 GPa	*E*_11_	96 GPa
*ν*	0.19	*ν*	0.38	*E*_22_	8.7 GPa
*α*	0.55 × 10^−6^/°C			*E*_33_	8.7 GPa
*ξ*	6.5 × 10^−6^/°C			*ν*_12_	0.30
*p*_11_	0.121			*ν*_13_	0.33
*p*_12_	0.270			*ν*_23_	0.38
*n*_eff_	1.468			*G*_12_	4.0 GPa
*K*_T_	10.79 pm/°C			*G*_13_	3.6 GPa
*K*_ε_	1.21 pm/με			*G*_23_	2.2 GPa
*r*	125 μm				
*L*	5 mm				

**Table 2:** Wavelength shift and separation of embedded FBG for each axial strain level.

Categories	Uniaxial Tension			Biaxial Tension		
	Δ*λ_avg_*/pm	*K_ε_^avg^* (pm/με)	|Δ*λ_diff_*|/pm	Δ*λ_avg_*/pm	*K_ε_^avg^* (pm/με)	|Δ*λ_diff_*|/pm
Case 1	560	1.223	3.4	−37.6	−1.051	101.0
Case 2	1120	1.223	6.7	−33.7	−0.848	101.1
Case 3	1680	1.223	10.1	−34.3	−0.872	101.2
Case 4	2239	1.223	13.4	−31.6	−0.783	101.3

**Table 3:** Modified STC matrix parameters for different biaxial tension cases.

Categories	STC_21_	STC_22_	STC_23_	STC_31_	STC_32_	STC_33_
Original	−0.123	0.093	0.009	−0.123	0.009	0.093
A	−0.123	0	0.009	−0.123	0.009	0.093
B-case1	−0.061	0	0.178	−0.123	0	0.178
B-case2	−0.061	0	0.228	−0.123	0	0.228
B-case3	−0.061	0	0.286	−0.123	0	0.286
B-case4	−0.061	0	0.328	−0.123	0	0.328
B-case4	−0.061	0	0.328	−0.123	0	0.328

## Data Availability

The original contributions presented in this study are included in the article/Supplementary Material. Further inquiries can be directed to the corresponding authors.

## Supplementary Materials

The following supporting information can be downloaded at: https://www.mdpi.com/article/10.3390/polym18151833/s1, S1. The impact of FBG position mismatch; Figure S1: Schematic diagram of the positions of the embedded FBG and the surface-mounted strain rosette; Figure S2: Contour plots of strain field components in CFRP under uniaxial tension at an applied axial strain of 2500 με, along with strain evolution at the FBG and strain gauge locations during the entire loading process; Figure S3: Strain transfer characteristics of the embedded FBG in CFRP under uniaxial tension: (a) strain components versus time; (b) FBG spectra; (c) wavelength shift versus axial strain.

## References

[1] Kwon H., Park Y., Kim J., Kim C. (2018). Embedded fiber Bragg grating sensor–based wing load monitoring system for composite aircraft. Struct. Health Monit..

[2] Yan D., Li Y., Zhou W., Qian Z., Wang L. (2025). A one-step integrated forming and curing process for smart thin-walled fiber metal laminate structures with self-sensing functions. J. Mater. Process. Technol..

[3] Air A., Gangadhara Prusty B. (2024). Manufacturing feasibility of a bend free ellipsoidal composite pressure vessel using automated fibre placement. Compos. Part A Appl. Sci. Manuf..

[4] Rajak D., Pagar D., Menezes P., Linul E. (2019). Fiber-Reinforced Polymer Composites: Manufacturing, Properties, and Applications. Polymers.

[5] Benazzo F., Rigamonti D., Bettini P., Sala G., Grande A.M. (2022). Interlaminar fracture of structural fibre/epoxy composites integrating damage sensing and healing. Compos. Part B Eng..

[6] Zhang Y., Wu X., Guo Q., Zhang D., Li C., Li D., Liu Y., Zhang J., Zhang P., Yan Y. (2025). Advances in sensors technologies for composites structural health monitoring. Compos. Struct..

[7] Todd M.D., Nichols J.M., Trickey S.T., Seaver M., Nichols C.J., Virgin L.N. (2007). Bragg grating-based fibre optic sensors in structural health monitoring. Philos. Trans. R. Soc. A Math. Phys. Eng. Sci..

[8] Yan G., Wan B., Huang H., Li W. (2024). Damage and Failure Monitoring of Aerospace Insulation Layers Based on Embedded Fiber Bragg Grating Sensors. Polymers.

[9] Ducoin A., Barber R.B., Wildy S.J., Codrington J.D., Baker A. (2023). Experimental evaluation of the use of embedded fiber Bragg gratings to measure steady and unsteady flow-induced marine propeller blade deformation. Ocean Eng..

[10] Zhu P., Feng X., Liu Z., Huang M., Xie H., Soto M.A. (2021). Reliable packaging of optical fiber Bragg grating sensors for carbon fiber composite wind turbine blades. Compos. Sci. Technol..

[11] Falcetelli F., Martini A., Di Sante R., Troncossi M. (2022). Strain Modal Testing with Fiber Bragg Gratings for Automotive Applications. Sensors.

[12] Liang Z., Liu D., Wang X., Zhang J., Wu H., Qing X., Wang Y. (2022). FBG-based strain monitoring and temperature compensation for composite tank. Aerosp. Sci. Technol..

[13] Luo G., Liou G., Xiao H. (2022). Using a Fiber Bragg Grating Sensor to Measure Residual Strain in the Vacuum-Assisted Resin Transfer Molding Process. Polymers.

[14] Minakuchi S., Niwa S., Takagaki K., Takeda N. (2016). Composite cure simulation scheme fully integrating internal strain measurement. Compos. Part A Appl. Sci. Manuf..

[15] Hu H., Li S., Wang J., Zu L., Cao D., Zhong Y. (2017). Monitoring the gelation and effective chemical shrinkage of composite curing process with a novel FBG approach. Compos. Struct..

[16] Shafighfard T., Mieloszyk M. (2022). Experimental and numerical study of the additively manufactured carbon fibre reinforced polymers including fibre Bragg grating sensors. Compos. Struct..

[17] Takeda S., Tsukada T., Sugimoto S., Iwahori Y. (2014). Monitoring of water absorption in CFRP laminates using embedded fiber Bragg grating sensors. Compos. Part A Appl. Sci. Manuf..

[18] Gabardi M., Tozzetti L., Faralli S., Solazzi M., Benedetti D., Rajbhandari S., Buttaro G., Di Pasquale F. (2023). Embedding Fiber Bragg Grating Sensors in Carbon Composite Structures for Accurate Strain Measurement. IEEE Sens. J..

[19] Sorensen L., Botsis J., Gmür T., Cugnoni J. (2007). Delamination detection and characterisation of bridging tractions using long FBG optical sensors. Compos. Part A Appl. Sci. Manuf..

[20] Takeda S., Minakuchi S., Okabe Y., Takeda N. (2005). Delamination monitoring of laminated composites subjected to low-velocity impact using small-diameter FBG sensors. Compos. Part A Appl. Sci. Manuf..

[21] Bosia F., Giaccari P., Botsis J., Facchini M., Limberger H.G., Salathé R.P. (2003). Characterization of the response of fibre Bragg grating sensors subjected to a two-dimensional strain field. Smart Mater. Struct..

[22] Lai M., Karalekas D., Botsis J. (2013). On the Effects of the Lateral Strains on the Fiber Bragg Grating Response. Sensors.

[23] Hu H., Li S., Wang J., Wang Y., Zu L. (2016). FBG-based real-time evaluation of transverse cracking in cross-ply laminates. Compos. Struct..

[24] Pereira G.F., Mikkelsen L.P., McGugan M., Kuzyk M.G. (2015). Crack Detection in Fibre Reinforced Plastic Structures Using Embedded Fibre Bragg Grating Sensors: Theory, Model Development and Experimental Validation. PLoS ONE.

[25] Guemes J.A., Menéndez J.M. (2002). Response of Bragg grating fiber-optic sensors when embedded in composite laminates. Compos. Sci. Technol..

[26] Hill D.J., Cranch G.A. (1999). Gain in hydrostatic pressure sensitivity of coatedfibre Bragg grating. Electron. Lett..

[27] Prabhugoud M., Peters K. (2006). Finite element model for embedded fiber Bragg grating sensor. Smart Mater. Struct..

[28] Albero Blanquer L., Marchini F., Seitz J.R., Daher N., Bétermier F., Huang J., Gervillié C., Tarascon J. (2022). Optical sensors for operando stress monitoring in lithium-based batteries containing solid-state or liquid electrolytes. Nat. Commun..

[29] Minakuchi S., Umehara T., Takagaki K., Ito Y., Takeda N. (2013). Life cycle monitoring and advanced quality assurance of L-shaped composite corner part using embedded fiber-optic sensor. Compos. Part A Appl. Sci. Manuf..

[30] Takagaki K., Minakuchi S., Takeda N. (2015). Thick-walled crack-free CFRP pipes: Stress reduction using atypical lay-up. Compos. Struct..

[31] Wachtarczyk K., Yadav N., Błachut A., Gąsior P., Schledjewski R., Kaleta J. (2024). Fatigue and residual strain monitoring for thermoplastic composite using embedded FBG inscribed in highly-birefringent side-hole elliptical core optical fiber. Measurement.

[32] Pereira G., McGugan M., Mikkelsen L.P. (2016). Method for independent strain and temperature measurement in polymeric tensile test specimen using embedded FBG sensors. Polym. Test..

[33] Montanini R., D Acquisto L. (2007). Simultaneous measurement of temperature and strain in glass fiber/epoxy composites by embedded fiber optic sensors: II. Post-cure testing. Smart Mater. Struct..

[34] Chen C., Wu Q., Zhang Y., Fan B., Xiong K. (2023). Multi-directional strain measurement under thermomechanical loading using embedded phase-shifted fiber Bragg grating. Measurement.

[35] Zhou C., Chen C., Ye Z., Wu Q., Xiong K. (2024). Multi-Directional Strain Measurement in Fiber-Reinforced Plastic Based on Birefringence of Embedded Fiber Bragg Grating. Sensors.

[36] Cao Z., Cao D., Li K., Li H., Cai W., Hu H., Li S. (2025). A novel mechanisms-based investigation on non-uniform curing fields and fiber–matrix interfacial behavior via self-sensing fibers. Compos. Struct..

[37] Roberts S.S., Davidson R. (1991). Mechanical Properties of Composites Materials Containing Embedded Fiber Optic Sensors. Fiber Optic Smart Structures and Skins IV.

[38] Kim K., Kollár L., Springer G.S. (1993). A Model of Embedded Fiber Optic Fabry-Perot Temperature and Strain Sensors. J. Compos. Mater..

[39] Martínez Vicente J.L., González-Gallego M., Terroba Ramírez F., Frövel M., Cela J.J.L. (2023). Study of the transverse strain effect on the Fiber Bragg Grating Sensor (FBGS) response with polyimide coating under experimental biaxial tests. Compos. Struct..

[40] Alemohammad H. (2018). Opto-Mechanical Modeling of Fiber Bragg Grating Sensors. Opto-Mechanical Fiber Optic Sensors.

[41] Erdogan T. (1997). Fiber grating spectra. J. Light. Technol..

[42] Hocker G.B. (1979). Fiber optic acoustic sensors with composite structure: An analysis. Appl. Opt..

[43] Mathews C.T., Sirkis J.S. (1990). Interaction Mechanics of Interferometric Optical Fiber Sensors Embedded in a Monolithic Structure.

[44] Pak Y.E. (1992). Longitudinal shear transfer in fiber optic sensors. Smart Mater. Struct..

[45] Kollar L.P., Van Steenkiste R.J. (1998). Calculation of the Stresses and Strains in Embedded Fiber Optic Sensors. J. Compos. Mater..

[46] Van Steenkiste R.J., Kollár L.P. (1998). Effect of the Coating on the Stresses and Strains in an Embedded Fiber Optic Sensor. J. Compos. Mater..

[47] González-Gallego M., Terroba Ramírez F., Martínez-Vicente J.L., González del Val M., López-Cela J.J., Frövel M. (2024). Fiber Bragg Gratings Sensor Strain–Optic Behavior with Different Polymeric Coatings Subjected to Transverse Strain. Polymers.

[48] Lammens N., Luyckx G., Voet E., Van Paepegem W., Degrieck J. (2015). Finite element prediction of resin pocket geometry around embedded optical fiber sensors in prepreg composites. Compos. Struct..

