# Supplementary material for: Investigation of Embedded FBG Response in Polymer Composites: Strain Transfer Mechanism and Interfacial Damage Under Multiaxial Loading
Source: Polymers (Basel). 2026 Jul 27;18(15):1833. doi: 10.3390/polym18151833 (PMC13468072; doi:10.3390/polym18151833)
Supplement: Supplementary file 1 [file polymers-18-01833-s001.zip › polymers-4426141-supplementary.pdf]

Supplementary Material

Investigation of Embedded FBG Response in Polymer Composites: Strain Transfer Mechanism and Interfacial Effect under Multiaxial Loading

Xingchen Yu[a,b], *Zihe Cao*[a,c]，*Dongfeng Cao*[a,c]，*Haixiao Hu*[a,b,c*]，*Hongda Chen*[a,b,c], *Wei Cai*[a,c], *Shuxin Li* [a, b,c*]

[a] *State Key Laboratory of Materials Synthesis and Processing, Wuhan University of Technology, Wuhan 430070, P. R. China*

[b] *National Energy Key Laboratory for New Hydrogen-ammonia Energy Technologies, Foshan Xianhu Laboratory, Foshan 528000, P. R. China*

[c] *Institute for Frontier and Interdisciplinary Innovation (School of Future Technology), Wuhan University of Technology, Wuhan 430119, China*

Correspondence:

Haixiao Hu, Email: yiming9008@126.com

Shuxin Li, Email: lishuxin@whut.edu.cn

S1. The impact of FBG position mismatch

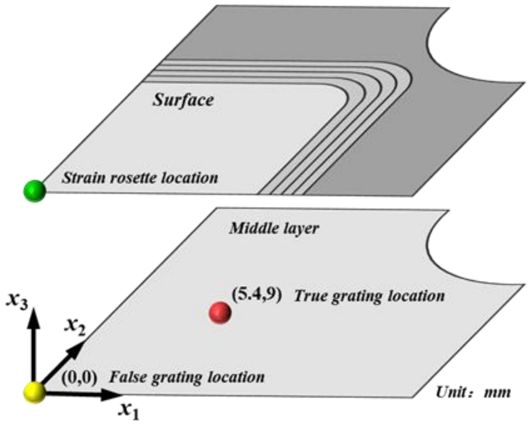

Figure. S1. Schematic diagram of the positions of the embedded FBG and the surface-mounted strain rosette

In the uniaxial tension test of Martínez et al. [S1], the calibrated axial strain sensitivity of the embedded FBG was 1.011 pm/με. This is lower than the theoretical K_ε

of 1.223 pm/με for a non-embedded FBG under the axial stress assumption, calculated from the fiber properties used in their study. Since uniaxial tension approximates an ideal axial stress state, this deviation is unexpected and thus merits further investigation.

A subsequent review of a related study [S2] revealed that the actual FBG embedding location was at coordinates (5.4, 9, 3), deviating from the initially reported specimen center (0, 0, 3), as shown in Figure S1.

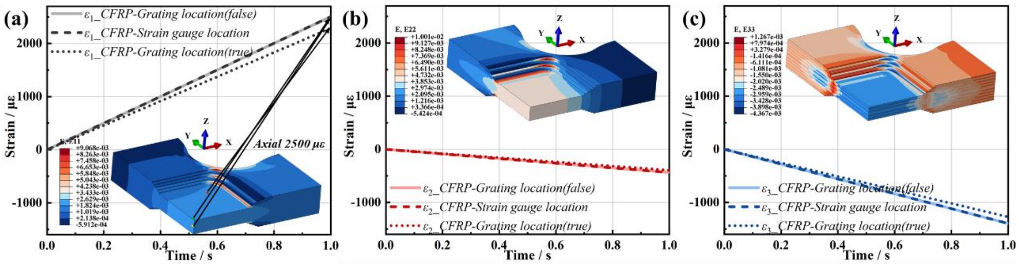

Figure S2. Contour plots of strain field components in CFRP under uniaxial tension at an applied axial strain of 2500 με, along with strain evolution at the FBG and strain gauge locations during the entire loading process.

To investigate the effect of this positional discrepancy, numerical simulations of the pristine CFRP laminate (without the embedded FBG) were performed under the experimental uniaxial tension condition. Strain components at three critical locations were extracted and compared: the originally reported FBG embedding position at the specimen center (0, 0, 3), the actual FBG embedding position at coordinates (5.4, 9, 3), and the surface strain rosette location (0, 0, 4.5). Contour plots of the strain field at an applied axial strain of 2500 με are shown, along with the evolution of strain components throughout loading (Figure S2).

The strain contour plots show a gradient in ε_1 within the *x*-*y* plane of the

center-thinned test region, while the through-thickness distribution remains uniform (Figure S2 (a)). This gradient means that the strain at the actual FBG location differs from that at the originally reported center, potentially affecting the sensitivity calibration. In contrast, the other two strain components are distributed relatively uniformly both in-plane and through the thickness (Figure S2(b-c)). Comparing the strain evolution curves at the three locations shows that the curves at the initially reported grating position and the strain rosette coincide. The curve at the actual grating location, however, deviates slightly (Figure S2(a)). This deviation is consistent with the strain gradient observed in the contour plots.

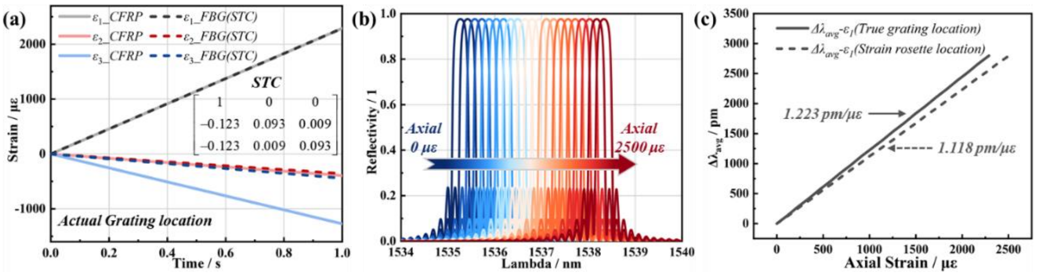

Figure S3. Strain transfer characteristics of the embedded FBG in CFRP under uniaxial tension: (a) strain components versus time; (b) FBG spectra; (c) wavelength shift versus axial strain.

The strain at the actual FBG embedding location in the CFRP was extracted and mapped to the FBG via the STC matrix (Figure S3(a)). The STC matrix was calculated from the material parameters of the CFRP host and the FBG reported by Martínez et al.[S1].

Throughout the uniaxial tension, $|\varepsilon_2 - \varepsilon_3|$ remained low. Because of this small strain difference, the FBG spectrum showed pure shift with no noticeable broadening, as shown in Figure S3(b). The wavelength shift of embedded FBG at (5.4, 9, 3) during

axial tension was plotted against the axial strain measured at two locations: the actual FBG embedding position (5.4, 9, 3) and the surface strain rosette location (0, 0, 4.5). The obtained K_ε from the actual FBG position was 1.223 pm/με for a non-embedded FBG. Using the surface strain rosette following the calibration procedure of Martínez et al. [S1] gave a K_ε^{avg} of 1.118 pm/με. This discrepancy explains why the axial strain sensitivity coefficient obtained by Martínez et al. [S1] from their uniaxial tension test was lower than the theoretical K_ε. If the FBG had been assumed to be embedded at (0, 0, 3) and calibrated using the axial strain at (0, 0, 4.5), where the two locations experience nearly identical axial strains. The resulting K_ε^{avg} would be 1.244 pm/με, a value closely approximates the theoretical K_ε.

In summary, while the K_ε^{avg} of an embedded FBG under axial stress is independent of its embedding location, experimental calibration requires strict spatial alignment between the reference strain point and the actual FBG position. Misalignment causes the strain measured at the reference point to deviate from that experienced by the FBG, leading to errors in the calibrated sensitivity.

[S1] Martínez Vicente JL, González-Gallego M, Terroba Ramírez F, Frövel M, Cela JJL. Study of the transverse strain effect on the Fiber Bragg Grating Sensor (FBGS) response with polyimide coating under experimental biaxial tests. Compos Struct 2023;326:117621. http://doi.org/10.1016/j.compstruct.2023.117621.

[S2] González-Gallego M, Terroba Ramírez F, Martínez-Vicente JL, González Del Val M, López-Cela JJ, Frövel M. Fiber Bragg Gratings Sensor Strain–Optic Behavior with Different Polymeric Coatings Subjected to Transverse Strain. Polymers (Basel) 2024;16(9):1223. http://doi.org/10.3390/polym16091223.
